# Photoluminescence mechanism of carbon dots: triggering high-color-purity red fluorescence emission through edge amino protonation

**DOI:** 10.1038/s41467-021-27071-4

**Published:** 2021-11-25

**Authors:** Qing Zhang, Ruoyu Wang, Bowen Feng, Xiaoxia Zhong, Kostya (Ken) Ostrikov

**Affiliations:** 1 State Key Laboratory of Advanced Optical Communication Systems and Networks, Key Laboratory for Laser Plasmas (Ministry of Education), School of Physics and Astronomy, Shanghai Jiao Tong University, Shanghai, 200240 China; 2 Institute of Molecular Medicine, Renji Hospital, School of Medicine, Shanghai Jiao Tong University, Shanghai, 200127 China; 3 School of Chemistry and Physics and QUT Centre for Materials Science, Queensland University of Technology (QUT), Brisbane, QLD 4000 Australia

**Keywords:** Materials science, Nanoscience and technology

## Abstract

Due to complex structure and surface functionalities, photoluminescence mechanisms of Carbon Dots are unknown, and it is challenging to synthesize Carbon Dots to achieve the desired optical properties. Herein, Carbon Dots simultaneously exhibiting high-color-purity (FWHM~24 nm) long wavelength one-photon fluorescence emission at 620 nm and NIR induced two-photon fluorescence emission at 630 and 680 nm are prepared by edge amino protonation treatment. Systematic analysis reveals that the protonation of 2,3-diaminophenazine changes the molecular state of Carbon Dots, decreases the photon transition band gap, and triggers red fluorescence emission with the dramatically narrowed peak width. As the oxidation products of reactant o-phenylendiamine, the emergence of 2,3-diaminophenazine as a photoluminescence determiner suggests that fluorophore products of precursor conversion are viable determinants of the desired luminescence properties of Carbon Dots. This work shows a new way for predicting and controlling photoluminescence properties of Carbon Dots, and may guide the development of tunable Carbon Dots for a broad range of applications.

## Abstract (web summary)

Carbon dots have garnered great interest due to their optical properties, however, engineering of the optical properties remains a challenge. Here, Zhang et al. produce carbon dots with a high color purity, providing a useful approach for engineering the optical properties of carbon dots.

## Introduction

Carbon dots (CDs), an emerging, highly-promising type of fluorescent carbon-based nanomaterials, have attracted tremendous research attention in diverse applications due to the fascinating merits of chemical inertness, excellent photon and thermal stability, high water solubility, and excellent biocompatibility^1–5^. Red fluorescence emission of CDs, especially the red fluorescence (FL) emission triggered by near-infrared (NIR, 800–1200 nm) excitation exhibits several advantages in biological applications because of low light absorption, weak auto-fluorescence, and deep-tissue penetration^6–11^.

However, due to the essentially unknown photoluminescence (PL) mechanism, most of the existing CDs require ultraviolet (UV) excitation, which may cause severe biological photo-damages and intense auto-fluorescence. The FL emission of the CDs is conventionally located in the blue−green light region showing shallow tissue penetration^12–15^. Moreover, practical applications of conventional CDs are also limited by the broad luminescence band with the full-width at half-maximum (FWHM) > 80 nm. This causes major issues to single out different FL signals during the spectral analysis in biological applications and the associated low-color-purity PL of photonic devices like LEDs^16,17^.

Intense efforts have been made to explore the inherent mechanisms controlling the wavelength red-shift of FL emission within CDs. By using complex separation approaches, previous research evidenced that the graphitic-N, −C−N, −C−O, and −COOH groups play a role in red-shifting one-photon PL emission (OPE) of CDs from blue to red emission for some CDs (Supplementary Table 1 of SI)^18–20^. However, due to complexities in compositions, surface functionalities, and crystal structures, there are few studies that could clearly identify the PL origin of CDs and further systematically analysis and demonstrate the relationship between the excitation state and the PL properties of the CDs^21,22^. It is now urgent to find reliable approaches for preparing long-wavelength (600−800 nm) red emission CDs with narrow-band luminescence, and to further reveal their PL mechanism. This knowledge is needed for the predictable and controllable synthesis of CDs with the desired PL features.

**Scheme 1:**
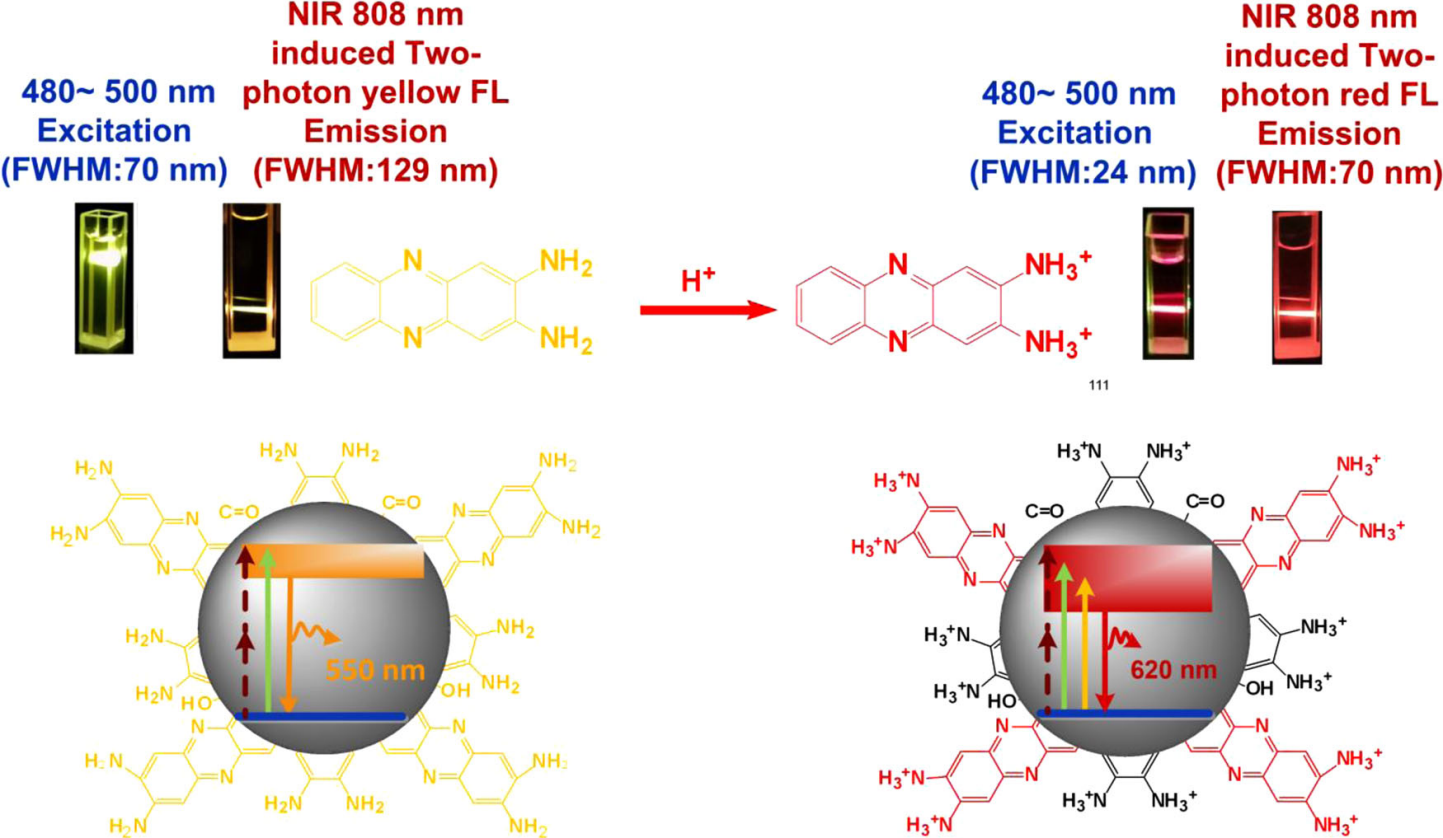
Mechanism of the red PL emission of the CDs. Protonation of surface 2,3-DAPN fluorophore strongly affects the molecular state of CDs, thus narrowing the transition band gaps and finally triggering one-photon and NIR excitation induced two-photon red fluorescence emission.

Herein, CDs simultaneously exhibiting one-photon red FL emission (620 nm) and NIR 808 nm induced two-photon red FL emission (630 and 680 nm) are prepared by protonating carbon dots derived from o-phenylendiamine (OPD). As shown in Scheme 1, the FWHM of one-photon red FL emission is narrowed down to 24 nm, which is 34% narrower compared to the latest major breakthrough showing the high-color-purity^16,17^. Systematic analysis demonstrates that protonation of the 2,3-diaminophe-nazine (2,3-DAPN) fluorophore decisively changes the molecular state of CDs, decreasing the photon transition bandgap, triggering one-photon and NIR induced two-photon high-color-purity red fluorescence emission. Furthermore, as 2,3-DAPN fluorophore is the OPD oxidation product, our identification of 2,3-DAPN as the CDs PL determiner suggests that fluorophore products of precursor conversion may serve as a predictor in the synthesis of CDs with the desired PL properties. Our work thus presents a new pathway for regulating and predicting high-color-purity red emission on CDs, which may act as a guide for developing high-performance CDs for diverse promising applications.

## Results and discussion

### Particle morphology and structure

**Fig. 1:**
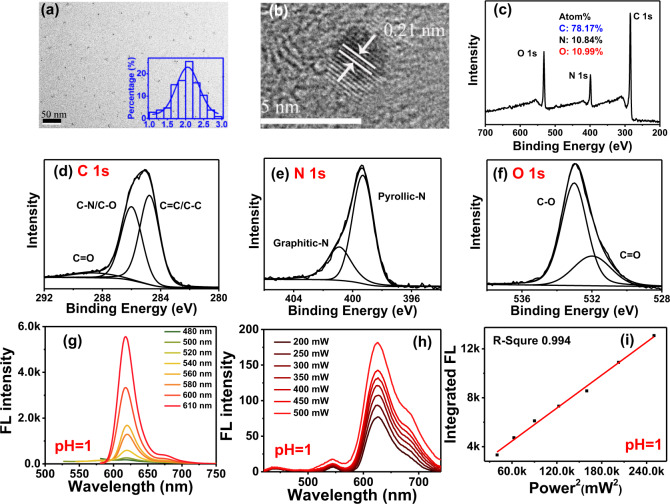
Structure, composition, and optical properties of protonated CDs. **a** TEM image of CDs (inset, particle size distribution of CDs); **b** high-resolution TEM image of CDs; **c**−**f**: full scale XPS spectra, high-resolution C 1s, N 1s, and O 1s XPS spectra of CDs; **g**, **h** one-photon and two-photon fluorescence (FL) spectra of protonated CDs in deionized water; **i** relationship of two-photon FL intensity and femtosecond (fs) laser power.

As shown in Fig. 1a, transmission electron microscope (TEM) images at 50 nm resolution demonstrate that the prepared CDs are well dispersed in aqueous solution. The inset image of Fig. 1a shows that the particle size of CDs is distributed in the range from 1 to 3 nm with the average dimension of 2 nm. High-resolution images presented in Fig. 1b indicate that CDs have a lattice distance of 0.21 nm attributed to the (1,0,0) plane of graphite. Raman spectrum of the CDs is recorded and shown in Supplementary Fig. 1. Vibration peaks presented at 1374 and 1607 cm^−1^ corresponding to the structure of *sp*^*3*^ (D) and *sp*^*2*^ (G), and the *I*_D_/*I*_G_ ratio of CDs is 0.80. The dominant strength of G peak indicates the graphitic structure nature of CDs in the study. The chemical composition of the prepared CDs is analyzed using X-ray photoelectron spectroscopy (XPS) analysis. As shown in Fig. 1c, results of full-scale XPS spectra analysis demonstrate that the prepared CDs consist of carbon (C), nitrogen (N), and oxygen (O) elements with atomic ratio of 78.17, 10.84, and 10.99%, respectively. High-resolution XPS spectra are shown in Fig. 1d–f. High resolution C 1s spectra (see Fig. 1d) reveal the presence of *sp*^2^ C=C carbon (284.7 eV), *sp*^*3*^ C−N/C−O carbon (286.0 eV) and C=O groups (288.6 eV). High-resolution N 1s spectra shown in Fig. 1e feature two peaks corresponding to pyrrolic nitrogen (399.3 eV) and graphitic nitrogen (400.9 eV). The O 1s band (see Fig. 1f) can be deconvoluted into two peaks located at 531.9 and 533.0 which represents the C=O, C−O−C/C−O−H groups^10,20^.

### PL property: one-photon and two-photon red fluorescence emission

The PL property of CDs can be strongly affected by the solvent environment. Thus, for better understanding the PL features of prepared CDs in biological water-soluble environment, the FL emission is measured by dissolving the protonated CDs samples in deionized water. As shown in Fig. 1g, when dissolved in deionized water, red FL emission of protonated CDs emerges at 620 nm with an FWHM of 24 nm which is 34% narrower than the latest breakthrough of high-color-purity CDs showing FWHM of 35 nm^16,17^. When excited by the wavelength varying from 480 to 610 nm, the prepared CDs exhibit an excitation-independent PL behavior. Experiments further demonstrate that, when excited by near-infrared (NIR) light of 808 nm femtosecond (fs) laser, up-conversion FL emission of CDs produces red emission lines at 630 and 680 nm with FWHM of 70 nm (see Fig. 1h). As shown in Fig. 1i, FL intensity of CDs increases according to the linear relationship (*R* = 0.994) with the square of laser power increase from 200 to 570 mW. This indicates that the red up-conversion FL of CDs belongs to two-photon excitation induced FL emission.

### PL evolution: H^+^ ions sensitive red FL emission

**Fig. 2:**
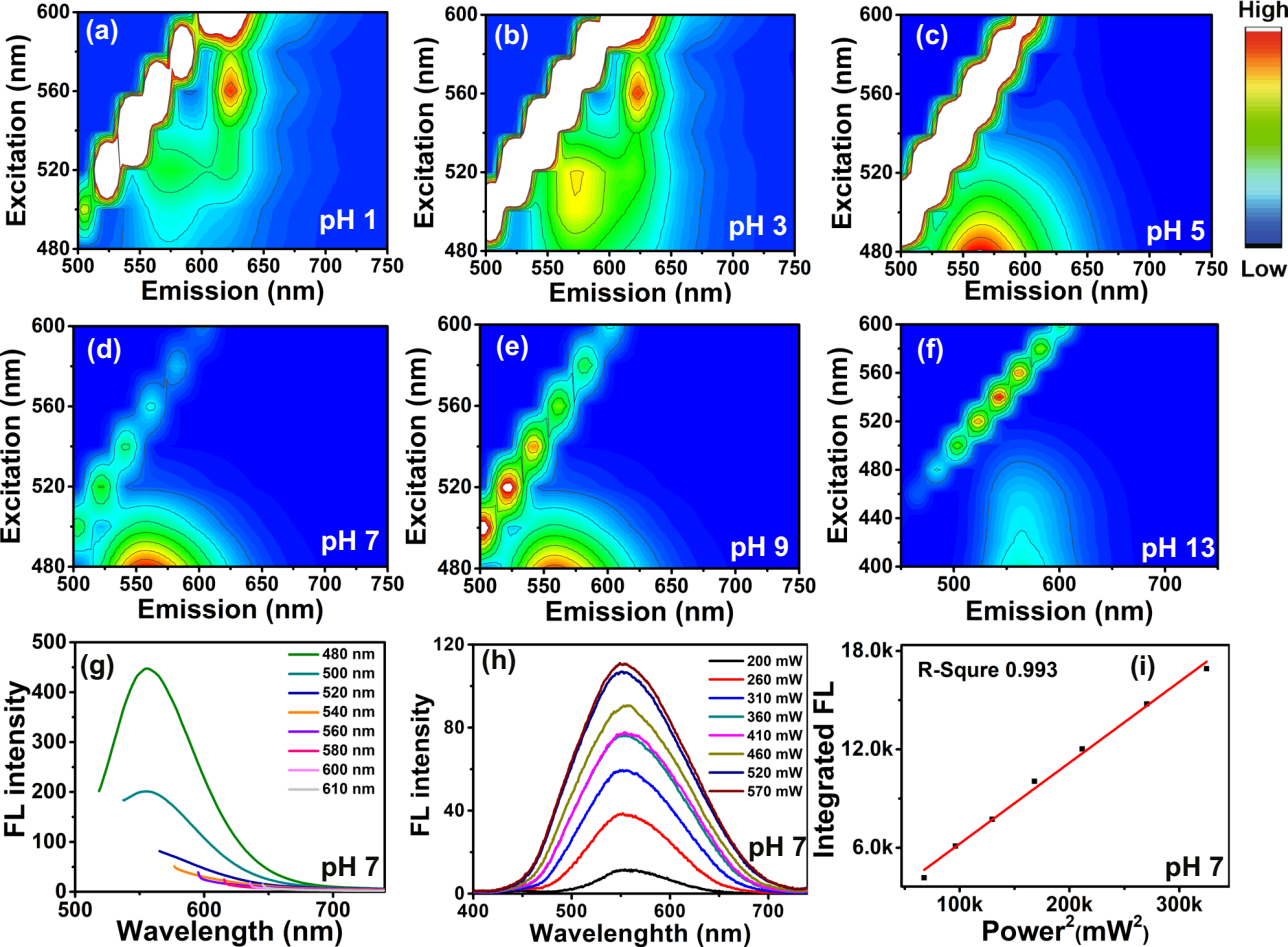
PL evolution of CDs under variable H^+^ ions concentration. **a**–**f** Normalized 3D FL spectra of CDs under different pH condition adjusted by H_2_SO_4_ and NaOH; **g**, **h** one-photon and two-photon fluorescence (FL) spectra of CDs without protonation treatment; **i** relationship of two-photon FL intensity and femtosecond (fs) laser power.

To investigate the effect of edge protonation on PL properties, 3-dimension (3D) FL spectra of CDs dissolved in varied acid-base conditions with pH ranging from 1 to 13 are recorded and the results are shown in Fig. 2a–f. Between pH 1 and pH 3 condition, CDs mainly exhibit red FL emission around 620 nm when excited by wavelength ranging from 500 to 600 nm. When pH increases from 5 to 14, FL emission of CDs is blue shifted and is located at 550 nm. It can be observed that that the prepared CDs mainly feature yellow FL emission (550 nm) in alkaline and neutral environments, while red PL emission behavior is exclusively triggered by the acidic condition.

Thus, for further comparison, detailed PL features of CDs at pH 7 are also recorded and are shown in Fig. 2g–i. As shown in Fig. 2g, under neutral conditions, CDs feature yellow one-photon fluorescence emission at 550 nm with FWHM of 70 nm when excited by light wavelength varying from 480 to 610 nm. Figure 2h demonstrates that the up-conversion FL emission of CDs at pH 7 excited by 808 fs laser are located at 550 nm with FWHM of 129 nm. Well-pronounced linear relationship (*R* = 0.993) between the up-conversion FL intensity and the square of 808 nm fs laser power (see Fig. 2i) indicates that two-photon FL emission also occurs during the up-conversion PL process at pH 7. In comparison with the PL behavior of the protonated CDs under pH 1 condition, with the decreases of pH, maximum one-photon fluorescence emission peak of CDs red-shifts from 550 to 620 nm. Two-photon fluorescence emission of CDs red-shifts from 550 nm towards 630 and 680 nm. In the meantime, FWHM of one-photon fluorescence narrows from 70 to 24 nm with a decrement of 66%, FWHM of two-photon fluorescence decrease from 129 to 70 nm with a decrement of 46%.

The current paradigm is that one-photon PL of CDs originates from either quantum confinement or surface or edge state effects, and two-photon PL of CDs was supposed to be in close relation with the specific electron structure, such as electron transition between π-conjugated carbon framework and electron-donating amine (−NH_2_) group^10,23,24^. In the case of this research, FL emission of CDs red-shifts along with the reducing pH, which indicates that the one-photon and two-photon PL may predominantly be controlled by the electron transition states of CDs significantly affected by the external H^+^ ion. Moreover, the excitation-independent one-photon PL behavior of CDs is similar to that of conventional organic dyes, implying that the FL emission may originate from the molecular fluorophores.

### Functional groups on CDs: residual −NH_2_ groups of OPD

To identify the fluorophore triggering red FL emission, surface functional group species on CDs are analyzed using Fourier transform infrared spectroscopy (FTIR), carbon and hydrogen nuclear magnetic resonance spectra (^13^C-NMR, ^1^H-NMR). To better understand the structure of CDs fluorophore of CDs, characteristic vibration spectra of o-phenylenediamine (OPD) are recorded and treated as a standard reference.

**Fig. 3:**
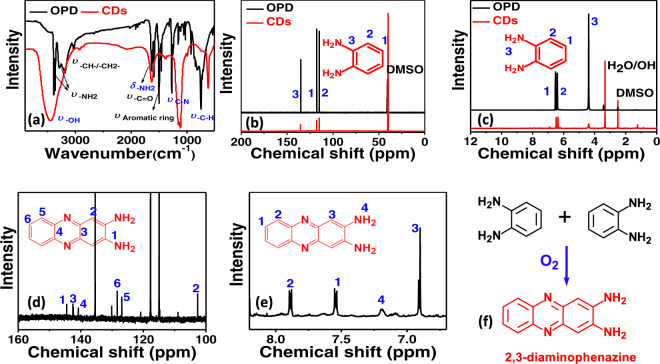
Identification of functional groups on CDs. **a**–**c** Fourier transform infrared spectroscopy (FTIR), ^13^C-NMR and ^1^H-NMR spectra of OPD and CDs; **d**, **e** zoomed ^13^C-NMR and ^1^H-NMR spectra of CDs. **f** Schematic showing the procedure of oxidizing OPD into 2,3-DAPN.

As shown in Fig. 3a, FTIR spectra of OPD feature two kinds of amino (−NH_2_) vibration bands located around 3367 and 3200 cm^−1^, respectively^25,26^. Due to the effect of hydrogen bond, −NH_2_ vibration around 3200 cm^−1^ is broadened about 100 nm. The stretching vibration of methylene groups (−CH−/−CH_2_−) is located at 3029 cm^−1^. The bending vibration of amino groups (−NH_2_) is around 1633 cm^−1^. Stretching vibration of the benzene ring is confirmed around 1462–1500 cm^−1^. In the case of CDs, in comparison with OPD, remarkable changes are seen in the decrease of −NH_2_ stretching vibration between 3200–3300 cm^−1^ and the significantly enhanced C-N stretching vibration around 1120 cm^−1^, which indicate that most of −NH_2_ groups of OPD may be converted into C−N−C structure during the thermal processes. FTIR spectra of CDs also reveal that bending vibration of −NH_2_ at 1633 cm^−1^ still exists, indicating that part of residual −NH_2_ groups of OPD may still be distributed on CDs after thermal process. Additionally, the emerging stretching vibration of hydroxyl (−OH) at 3450 cm^−1^ evidences that new kinds of organic functional groups are generated on CDs.

The results of ^13^C-NMR spectroscopy are shown in Fig. 3b. OPD features three kinds of chemical shifts located at 135, 115, and 117 ppm^27,28^. Among these peaks, the chemical shift at 135 ppm is caused by carbon vibration in C−N structures, whereas the chemical shifts around 115 and 117 ppm are attributed to C−C/C=C vibration of benzene ring. As shown in Fig. 3b, CDs show the same chemical shift at 135, 115, and 117 ppm, which indicates that residual −NH_2_ groups of OPD are preserved after pyrolysis and are distributed on CDs.

^1^H-NMR spectra are presented in Fig. 3c. In the case of OPD, hydrogen vibration of the benzene ring is demonstrated at 6.4 and 6.5 ppm, chemical shift of hydrogen within −NH_2_ is located at 4.38 ppm^27,29^. In agreement with the results of ^13^C-NMR analysis, CDs feature identical chemical shifts of hydrogen at 6.4, 6.5, and 4.38 ppm in comparison with that of OPD. These evidences further confirm the distribution of residual −NH_2_ groups of OPD on CDs. Meanwhile, it can also be observed that the vibration strength of hydrogen within −NH_2_ dramatically decreases, which is in accordance with the above FTIR results. Collectively, the results of FTIR,^13^C-NMR, and ^1^H-NMR analyses reveal that the residual −NH_2_ groups of OPD are predominantly distributed on the surface of CDs.

### Functional groups on CDs: New 2,3-DAPN fluorophore oxidized from OPD

By zooming up the details of NMR spectra (see Fig. 3d, e and Supplementary Figs. 2 and S3), we also find that new chemical shifts belonging to fluorophore 2,3-diaminophenazine (2,3-DAPN) emerge on the surface of CDs. As shown in Fig. 3d, characteristic chemical shifts of ^13^C-NMR spectra at 102.6, 128.3, 126.8, 144.0, 142.6, and 140.7 ppm are in accordance with that of 2,3-DAPN demonstrated by previous publications (see Supplementary Fig. 4)^30^. Among the ^13^C-NMR spectra, peaks located at 144.0, 142.6, 140.7 ppm corresponds to carbon vibration within C−NH_2_ and C−N=C structure of 2,3-DAPN, respectively. Chemical shifts of 128.3, 126.8, and 102.6 ppm are caused by C−C/C=C vibration within the aromatic ring. Zoomed ^1^H-NMR spectra are shown in Fig. 3e. Chemical shifts located at 7.8, 7.5, and 6.9 ppm caused by hydrogen vibrations within benzene ring, and chemical shift at 7.2 ppm attributed to active hydrogen vibration of −NH_2_ are in a good agreement with the corresponding shifts of 2,3-DAPN reported previously (see Supplementary Fig. 5)^30^. Additionally, other organic functional groups including carboxyl (−COOH), methylene (−CH_2_−), and aldehyde (−CHO) also can be observed within both zoomed ^13^C-NMR and ^1^H-NMR spectra (see Supplementary Figs. 2 and  3).

Therefore, in summary, combined with the results of FTIR, ^13^C-NMR, and ^1^H-NMR analysis, surface functional groups of CDs are dominated by residual −NH_2_ groups of OPD with the low abundance of 2,3-DAPN, −OH, −COOH, −CH_2_−, and −CHO groups. As evidenced by previous findings, 2,3-DAPN is an oxidation product of OPD. Thus, it can be concluded that oxidative polymerization between OPD molecules has occurred (see Fig. 3f) during the process of preparation, and the products of oxidation are further distributed on CDs.

### Protonation induced fluorophore evolution: −NH_2_ → −NH${ }_{3}^{+}$

To investigate the structure evolution of surface functional group under protonation treatment, characteristic vibration spectra of CDs before and after H^+^ treatment are detected and compared using FTIR, NMR, and Zeta potential analysis.

**Fig. 4:**
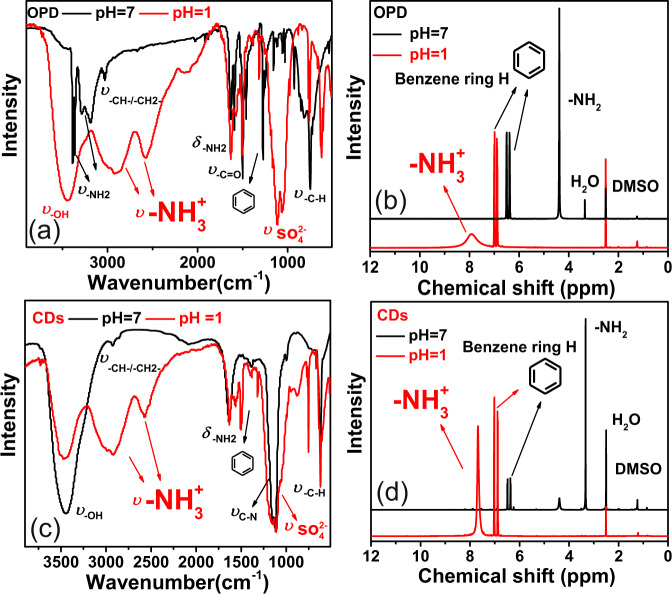
Structural evolution of functional groups during protonation treatment. **a**, **b** FTIR and ^1^H-NMR spectra of OPD with or without protonation treatment. **c**, **d** FTIR and ^1^H-NMR spectra of CDs with or without protonation treatment (black line: spectra of samples without protonation treatment; red line: spectra of samples with protonation treatment).

As a reference, characteristic spectra evolution of OPD under protonation treatment are firstly measured and analyzed. As the FTIR spectra shown in Fig. 4a, after protonation treatment, amino (−NH_2_) vibration of OPD at 3367 and 3200 cm^−1^ disappear, while new dispersive peaks attributed to ammonium (${-{{{{{\rm{NH}}}}}}}_{3}^{+}$) stretching vibration emerge at 2889 and 2586 cm^−1^^31^. Characteristic vibration of ${{{{{{\rm{SO}}}}}}}_{4}^{2-}$ groups are revealed around 1100 cm^−1^^32^. ^1^H-NMR spectra evolution of OPD during protonation is presented in Fig. 4b. It can be observed that, after protonation, chemical shifts of benzene ring are shifted and relocated at 7.0 and 6.87 ppm with an increment of 0.5 ppm. Chemical shift of amino (−NH_2_) vanishes, new chemical shift belongs to ammonium (${-{{{{{\rm{NH}}}}}}}_{3}^{+}$) vibration emerges at 7.91 ppm^33^. Thus, the results of FTIR and ^1^H-NMR analyses evidence that, due to intense protonation, when amino (−NH_2_) groups of OPD encounter H^+^ ions, functional groups of −NH_2_ will be converted into the form of ${-{{{{{\rm{NH}}}}}}}_{3}^{+}$.

Characteristic spectra evolution of CDs is presented in Fig. 4c, d. As shown in Fig. 4c, FTIR analysis results of CDs are in accordance with the corresponding analysis of OPD. When protonated at pH 1, dispersive vibration bands of ${-{{{{{\rm{NH}}}}}}}_{3}^{+}$ groups emerge at 2932 and 2576 cm^−1^. Due to the overlaying vibration of ${{{{{{\rm{SO}}}}}}}_{4}^{2-}$and C−N groups, vibration bands of CDs around 1100 cm^−1^ are broadened and enhanced. The evolution of ^1^H-NMR spectra is shown in Fig. 4d. In agreement with the corresponding spectra of OPD, the chemical shift of amino (−NH_2_) within CDs completely disappeared, new chemical shift belongs to hydrogen atom vibration within ${-{{{{{\rm{NH}}}}}}}_{3}^{+}$emerges at 7.68 ppm. The zeta potential analysis further demonstrated that, after protonation treatment, the surface potential of CDs increases from −8.32 to 38 mV, which confirms the transformation of −NH_2_ groups into groups ${{\mbox{-N}}}{{{\mbox{H}}}}_{3}^{+}$.

Collective results of FTIR, ^13^C-NMR, ^1^H-NMR, and zeta potential indicate that, when treat with pH condition adjusted by H_2_SO_4_, surface functional groups of CDs are effectively protonated, and the abundant −NH_2_ groups on the CDs surface are converted into ${{\mbox{-N}}}{{{\mbox{H}}}}_{3}^{+}$. It is clear that the red FL emission is closely related with the acidity of the environment. Therefore, it can be concluded that protonation of functional groups containing −NH_2,_ such as residual -NH_2_ groups of OPD and 2,3-DAPN may determine the red FL emission of CDs.

Based on the above results, protonation of −NH_2_ into ${-{{{{{\rm{NH}}}}}}}_{3}^{+}$ groups on CDs surface may indeed induced the red FL emission with high probability. According to the analysis results of surface functional groups, the residual amino (−NH_2_) group of OPD and new emerged 2,3-DAPN on CDs are the only two fluorophores that contain the −NH_2_ functional groups. Therefore, molecular monomers of OPD and 2,3-DAPN were purchased from the company and the PL characteristic spectra were detected and compared with that of CDs.

### 2,3-DAPN dominating visible light absorption

UV-vis absorption of OPD, 2,3-DAPN, and CDs at pH 7 are shown in Supplementary Fig. 6a. OPD (black line) features two absorption peaks located at 240 and 287 nm due to π→π* transition of the benzene ring. Non-absorption peaks of OPD are observed in the visible light region between 400 and 750 nm. 2,3-DAPN (red line) exhibits the π→π* transition absorption band at 258 nm. The n→π* transition of 2,3-DAPN is characterized by the absorption peak around 420 nm. Absorption bands of CDs at 242 nm and 281 nm are caused by π→π* transition of C−C/C=C structure, and the absorption peaks around 420 nm are attributed to the n→π* transition. In comparison with that of OPD and 2,3-DAPN, CDs display the same visible light absorption band with the absorption band of 2,3-DAPN, and no similar absorption bands are observed in the spectra of OPD shown in the visible light region at pH 7.

UV-Vis absorption spectra of OPD, 2,3-DAPN, and CDs at pH 1 are presented in Supplementary Fig. 6b. When encountered with strongly acidic conditions of pH 1, the OPD (black line) still exhibits the two absorption bands located at 240 and 287 nm and has no visible light absorbance. Indifferent with that of pH 7 condition, the absorption intensity of OPD at 287 nm sharply decreases due to the protonation of amino group (−NH_2_). In the case of 2,3-DAPN (red line of Supplementary Fig. 6b), absorption bands of UV light region are revealed at 260 and 270 nm, and visible light absorption is red-shifted and relocated at 465 and 485 nm. At pH 1 condition, CDs (blue line of Supplementary Fig. 6b) features π→π* transition absorption locating at 260 and 270 nm, and n→π* transition relocated at 465 and 485 nm which is in well accordance with the absorption spectra of 2,3DAPN.

Therefore, the evidences of UV−Vis absorption reveal that 2,3-DAPN features a more similar absorption behavior with the CDs than that of OPD in visible light region, which implies that the 2,3-DAPN fluorophore may dominate the electron transition process of CDs during the visible light excitation for red fluorescence-emission.

### Protonated 2,3-DAPN triggering one-photon red FL

**Fig. 5:**
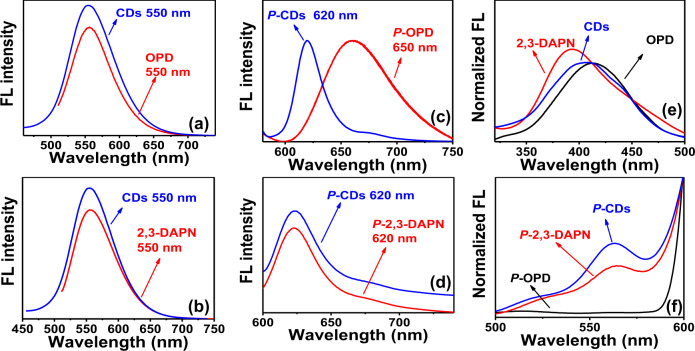
PL comparison between the fluorophore monomer and CDs. **a**, **b** FL emission of CDs, OPD, and 2,3-DAPN under the excitation of 480 nm; **c**, **d** FL emission of protonated CDs, protonated OPD, and protonated 2,3-DAPN under the excitation of 560 nm. **e** Excitation spectra of OPD, 2,3-DAPN, and CDs with FL emission located at 550 nm; **f** excitation spectra of protonated CDs, OPD, and 2,3-DAPN featuring FL emission at 620 nm. (P-CDs: CDs with protonation treatment, P-OPD, and P-2,3-DAPN: OPD and 2,3-DAPN molecular at pH 1 condition).

One-photon FL spectra of OPD and 2,3-DAPN are shown in Fig. 5a, b. When excited with the wavelength at 520 nm, the OPD and 2,3-DAPN both feature the FL emission peak at 550 nm. These yellow FL emission peaks of OPD and 2,3-DAPN are consistent with the corresponding peaks of CDs, indicating that residual −NH_2_ groups of OPD and 2,3-DAPN may both be involved in the generation of the yellow FL emission of the CDs.

Due to the rapid oxidation of OPD in the water (see Supplementary Fig. 7), the protonated CDs are compared with the OPD and 2,3-DAPN that are dissolved at the pH 1 condition. The one-photon fluorescence (FL) spectra of protonated CDs, protonated OPD, and protonated 2,3-DAPN are recorded and displayed in Fig. 5c, d. As shown in Fig. 5c, when excited by the wavelength of 560 nm, protonated OPD exhibits the red FL emission located around 650 nm with full width at half maximum (FWHM) of 68 nm. In comparison to the protonated CDs (620, 24 nm), the FL emission of protonated OPD is red-shifted about 38 nm with a broadened FWHM. In the case of protonated 2,3-DAPN, (shown in Fig. 5d), the FL emission peak is around 620 nm with FWHM of 24 nm which is very close with that of the protonated CDs

Furthermore, as shown in Fig. 5e, the FL excitation peak of OPD, 2,3-DAPN, and CDs are located around 414, 395, and 410 nm, corresponding to FL emission at 550 nm. The similarities presented between the excitation wavelength further confirm that −NH_2_ residues of both OPD and 2,3-DAPN are involved in yellow FL emission of CDs. As shown in Fig. 5f, after protonation treatment, for red FL emission at 620 nm, the FL excitation peaks of protonated OPD, protonated 2,3-DAPN and protonated CDs are locating at 512, 560, and 560 nm. No similarities are observed in the excitation spectra between the protonated OPD and protonated CDs, and the identical excitation peaks are present within the excitation spectra of protonated 2,3-DAPN and protonated CDs.

Thus, it can be concluded that CDs feature similar one-photon FL emission and excitation behavior with the 2,3-DAPN molecular, while only a few similarities can be found in the optical properties of OPD. As such, these results collectively confirm that the emerged 2,3-DAPN fluorophore on CDs may have dominated the CDs one-photon PL mechanism, and triggered the one-photon red FL emission after protonation.

Additionally, our experimental results also reveal that differences also exist between PL properties of 2,3-DAPN and CDs. The obtained PLQYs of CDs, OPD, and 2,3-DPNA in different pH conditions are shown in Supplementary Table 2. FL QYs of CDs is 14% in the neutral environment, and when protonated under the acidic condition, the FL QYs of red emission is 1.9%. Similar with that of CDs, fluorophore 2,3-DAPN shows a QYs of 24% in neutral condition and 0.2% after protonation. These differences suggest that the interaction between the 2,3-DAPN and the carbon framework also affects the FL emission of CDs. In the case of OPD, due to nearly non-absorbance (less than 0.001) in the visible light region (see Supplementary Fig. 8a,  b), it is difficult to measure the QYs. To get a simple comparison, FL emission of OPD, 2,3-DAPN, and CDs with equal mass concentration of 125 μg·mL^−1^ are further detected at the neutral environment and after protonation. As shown in Supplementary Fig. 8c, the FL intensity of 2,3-DAPN and prepared CDs are 48 and 7.7 times higher than that of OPD at pH 7. FL intensity of 2,3-DAPN and CDs is 2.45 and 2.8 times higher than that of OPD after protonation (see Supplementary Fig. 8d). These results may indicate that the OPD features the identical FL emission peak at 550 nm compared with the emission of CDs at the neutral environment and exhibits different FL emission peaks with the CDs located at 650 nm after protonation. However, due to the low FL intensity of OPD compared with that of 2,3-DAPN, the one-photon FL behavior of CDs may still be determined by the molecular state of 2,3-DAPN fluorophore.

### Protonated 2,3-DAPN triggering two-photon red FL

Up-conversion FL emission of 2,3-DPNA excited by 808 fs laser is detected and shown in Supplementary Fig. 9a. 2,3-DPN features FL emission peak located at 550 nm with full width at half maximum (FWHM) of 121 nm. A well-pronounced linear relationship (*R* = 0.991) between the FL intensity and the square of fs laser power at 808 nm (see Supplementary Fig. 9b) indicates that two-photon FL emission occurs during the up-conversion PL process. As presented in Supplementary Fig. 9c, up-conversion FL emission of protonated 2,3-DPNA excited by 808 fs laser appears around 629 nm with FWHM of 76.4 nm. The linear relationship (*R* = 0.995) between the FL intensity and the square of 808 nm fs laser power (see Supplementary Fig. 9d) demonstrates that the up-conversion FL emission of protonated 2,3-DPNA can be attributed to the two-photon FL emission.

Up-conversion FL emission of OPD excited by the 808 nm fs laser is detected and shown in Supplementary Fig. 10a. The maximum FL emission peak of OPD is located at 550 nm, and the FWHM is 129 nm. The well-pronounced linear relationship (*R* = 0.995) between the FL intensity and the square of 808 nm fs laser power (see Supplementary Fig. 10b) is also found and further confirms that two-photon FL emission occurs during the up-conversion PL process. Up-conversion FL emission of protonated OPD excited using 808 nm fs laser is detected and is shown in Supplementary Fig. 10c. The maximum FL emission peak of OPD is located at 662 nm, and the FWHM is 68.54 nm. The linear relationship (*R* = 0.999) between the FL intensity and the square of the laser intensity (see Supplementary Fig. 10d) shows that two-photon FL emission occurs during the up-conversion PL process.

**Fig. 6:**
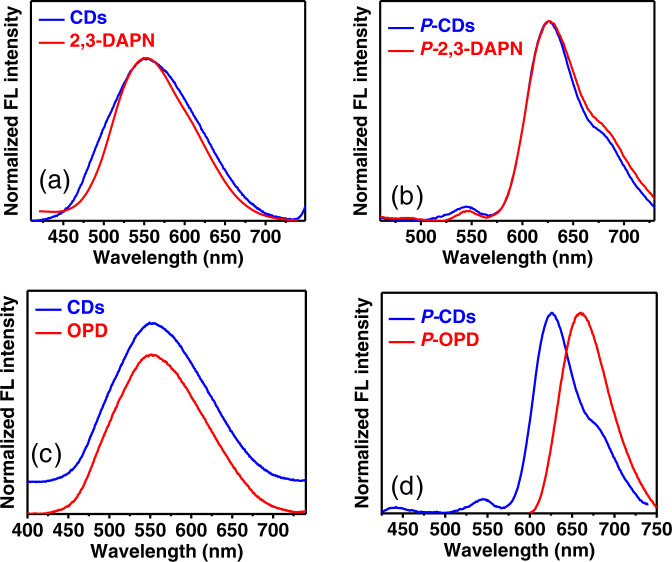
Comparison of two-photon emission (TPE) between the 2,3-DAPN, OPD, and CDs. **a** TPE of 2,3-DAPN and CDs without protonation treatment, **b** TPE of 2,3-DAPN and CDs with protonation treatment; **c** TPE of OPD and CDs without protonation treatment, **d** TPE of OPD and CDs with protonation treatment; (P-CDs: CDs with the protonation treatment, P-OPD, and P-2,3-DAPN: OPD and 2,3-DAPN molecular at pH 1 condition).

To investigate the mechanism of the two-photon PL emission, two-photon features of OPD, 2,3-DAPN, and CDs are normalized in Fig. 6 and further compared. In the case of 2,3-DAPN (see Fig. 6a, b), two-photon FL emission of 2,3-DAPN without protonation is located at 550 nm with the FWHM of 121 nm. The two-photon FL emission of 2,3-DAPN with protonation emerges at 629 nm with the FWHM of 76.4 nm. In comparison with that of CDs, 2,3-DAPN features the similar tow-photon maximum emission peak and the similar FWHM in both conditions. As shown in Fig. 6c, unprotonated OPD exhibits two-photon FL emission at 550 nm, which is consistent with the emission of unprotonated CDs. After the protonation treatment, (see Fig. 6d), the two-photon FL emission of protonated OPD red-shifts into 662 nm and is different from the emission of protonated CDs appearing at 630 nm. Thus, based on the above results, it can be concluded that 2,3-DAPN and OPD may both be involved in the two-photon emission of CDs before the protonation. While after protonation treatment, protonated 2,3-DAPN determines the two-photon PL of CDs.

Therefore, the above results related to the UV−Vis absorption, one-photon, and two-photon FL emission collectively confirm that the 2,3-DAPN fluorophore features more similar optical properties with the CDs compared to OPD. In other words, the emerged 2,3-DAPN fluorophore on CDs may have dominated the CDs PL mechanism and triggered the red FL emission after the protonation induced by H^+^ treatment.

### Molecular state of CDs PL determined by 2,3-DAPN fluorophore

To understand whether the molecular state of 2,3-DAPN fluorophore indeed exists in the CDs system, CDs products are further separated by silica column chromatography. The adsorbed CDs are eluted using organic solution by mixing ethyl acetate and petroleum ether in the volume ratio of 4:1, and then the organic solution is removed by rotary evaporation. After column chromatography, the optical property of CDs is recorded and compared with that of the CDs purified using dialysis (see Supplementary Fig. 11). As shown in Supplementary Fig. 11a, the CDs purified using silica column chromatography are highly monodisperse in the water. Supplementary Fig. 11b illustrates that the interplanar spacing of the prepared CDs is 0.21 nm. Supplementary Fig. 11c shows that the average particle diameter of CDs after the column chromatography is around 1.5 nm. UV−vis absorption of CDs with or without protonation treatment are shown in Supplementary Figs. 11d and 11g, respectively. The visible absorption band of CDs treated with column chromatography and dialysis are both located around 420 nm. FL emission of both kinds of CDs (see Supplementary Fig. 11e) are revealed and shown at 550 nm. Once being protonated (Supplementary Fig. 11h), the FL emission of CDs is red-shifted to 620 nm. It can be observed that FL spectra of CDs purified with column chromatography and dialysis are in accordance with each other. The FL excitation spectra of both CDs are measured and shown in Supplementary Fig. 11f–i. The results of Supplementary Fig. 11f show that for FL emission at 550 nm, the excitation band of CDs ranges from 350 to 500 nm. Supplementary Fig. 11i indicates that, for protonated CDs with emission at 620 nm, the excitation bands varied between 500 and 600 nm. The excitation spectra of the CDs purified using dialysis and column chromatography overlap well with each other.

The results of the comparison demonstrate that CDs obtained using column chromatography feature similar optical properties compared with the CDs purified using dialysis. As the CDs purified using dialysis have similar optical features with the fluorophore of 2,3-diaminophenazine (2,3-DAPN), therefore, this result further supports that molecular state fluorophore of 2,3-DAPN indeed exists in the system and dominates the one-photon red emission of CDs.

To understand the effect of carbonization degree on the transformation of molecular state and carbon core state, we prepared CDs with different carbonization degree by heating at temperature varied from 60 to 220 °C. As shown in Supplementary Fig. 12a–d, the CD samples prepared at temperatures 60, 120, 180, and 220 °C all feature the similar Raman vibration peak around 1400 and 1600 cm^−1^, which correspond to *sp*^*3*^ (D) and *sp*^*2*^ (G) hybridization that determine the carbonization degree of the CDs. As the strength of G peak indicates the graphitic structure, thus the ratio of *I*_D_/*I*_G_ can be used to quantify the carbonization degree. As shown in Supplementary Fig. 12a–d, the *I*_D_/*I*_G_ ratio are 1.15, 0.99, 0.84, and 0.8 for samples prepared at 60, 120, 180, and 220 °C, which indicate that CDs samples with the gradually increasing carbonization degree are obtained.

Therefore, to investigate the effect of the carbonization degree on the transformation of the molecular state and carbon core state, the FL emissions of CDs are measured and compared with each other. As shown in Supplementary Fig. 12e, FL emission of CDs with different carbonization degree appears at 550 nm. When being protonated (see Supplementary Fig. 12f), the FL emission of all the CD samples is red-shifted to 620 nm. A very weak difference is observed between the PL behavior of CDs prepared at 60, 120, 180, and 220 °C. As the molecular state of fluorophore 2,3-DAPN dominates the PL behavior of CDs prepared at 220 °C, we conclude that the carbonization degree has only a weak effect on the transformation of the molecular state.

Based on the results of silica column chromatography and the investigation of the effect of carbonization degree on the transformation of the molecular state and carbon core state, it can be concluded that the PL of CDs  is determined by the molecular state of 2,3-DAPN fluorophore once it is formed in the beginning of generation.

### Internal energy level evolution during edge protonation

**Fig. 7:**
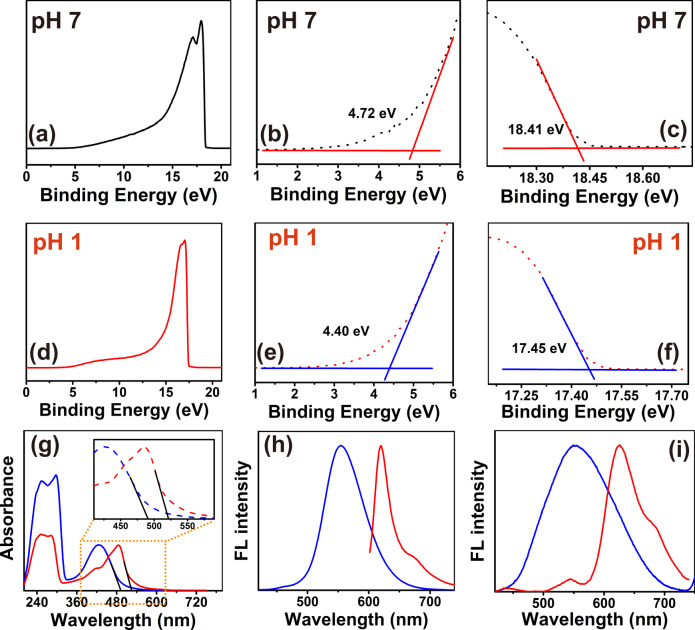
Energy level structure variation of the CDs by the protonation treatment. **a**−**c** full-scale ultraviolet photoelectron spectroscopy (UPS), near Fermi energy (low binding energy) and secondary electron cut-off (high binding energy) regions of CDs before the protonation; **d**−**f** UPS, near Fermi energy and secondary electron cut-off regions of CDs after the protonation. **g** UV−Vis spectra, **h** one-photon fluorescence spectra; **i** two-photon fluorescence spectra of CDs before and after the protonation. (P-CDs: CDs after the protonation treatment).

To reveal the detailed internal energy level structure of the carbon dots, the HOMO energy level of CDs before and after protonation are both analyzed using ultraviolet photoelectron spectroscopy (UPS). Full scale UPS spectra are shown in Fig. 7a, d. Near Fermi energy and secondary electron cut-off regions of CDs are presented in Fig. 7b, c, e, f. The HOMO level of CDs is directly calculated from the UPS data according to the following data following the procedure described elsewhere (see “Methods” section)^34^. As the energy of the incident photons *E*_Incident photon_ is 21.2 eV here, the HOMO energy level of CDs is calculated to be 7.25 and 8.15 eV before and after the protonation, respectively.

As shown in Fig. 7g, the absorption edges of CDs before and after the protonation treatment are located at 490 and 521 nm, respectively. Based on the relationship between the forbidden bandwidth and UV−vis absorption edge *E*_g1_ = 1240/*λg*, the energy gap of unprotonated CDs and protonated CDs between the HOMO and LOMO energy level are calculated as 2.53 and 2.38 eV. Figure 7h, i shows that the FL emission peak of unprotonated CDs is located at 550 nm, while when subjected to the protonation treatment, the maximum one-photon fluorescence emission peak red-shifts to 620 nm and the maximum two-photon fluorescence emission peak relocates to 630 nm. Thus, the bandgap for the FL emission before and after protonation are 2.26 eV (550 nm), and 2.0 eV (620 nm) according to equation *E*_g_ = *hν*.

**Fig. 8:**
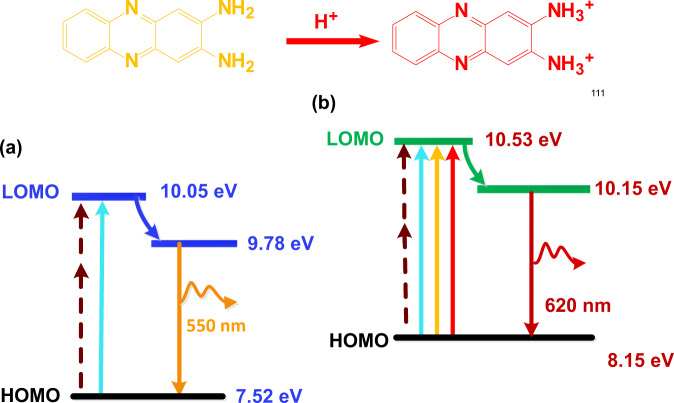
Internal energy level variation of CDs by the protonation treatment. **a** CDs without the protonation; **b** CDs treated with the edge protonation.

Thus, the detailed internal energy level structure of the carbon dots before and after protonation and the changes in the electronic transition process are revealed and shown in Fig. 8. The protonation treatment increases the HOMO energy level of CDs from 7.52 to 8.15 eV, decreases the energy gap between the HOMO and LOMO from 2.53 to 2.38 eV, and narrows the energy gap of FL emission from 2.26 to 2.0 eV.

Therefore, combined with the results revealed by fluorophore structure analysis, fluorophore state evolution during protonation, and PL origin identification, it can be concluded that the protonation of 2,3-DAPN fluorophore has changed the PL state of CDs, which rises the internal HOMO energy level of the CDs and decreases the energy gap between the HOMO and LOMO energy level, then eventually triggering red-shifted one-photon and two-photon FL emission.

## Applications

Due to the interesting optical properties, the prepared CDs are further applied in diverse applications including fluorescent labeling of *Escherichia coli* bacteria, and in vivo imaging of mice.

### Fluorescence bio-labeling of *Escherichia coli*

**Fig. 9:**
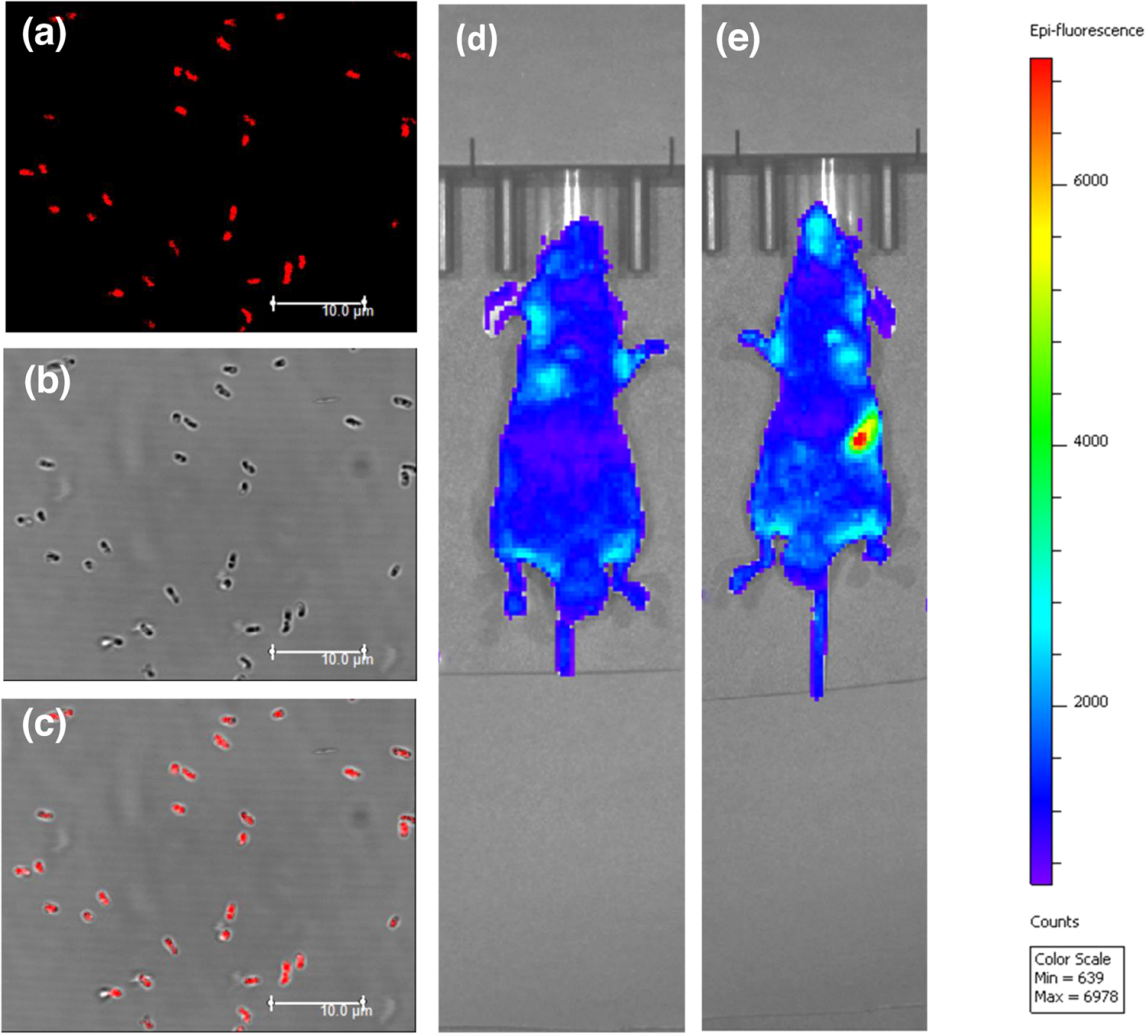
Applications of CDs. **a**–**c** Confocal images of *E. coli* treated with protonated CDs: **a** confocal FL images; **b** bright field images; **c** merged picture of (**a**) and (**b**); **d** in vivo fluorescence images of the mouse before gavage injection; **e** in vivo fluorescence images of the mouse after gavage injection.

In fluorescence bio-imaging application, *Escherichia coli* are cultured and treated with protonated CDs at 37 °C for 6 h. Confocal fluorescence images of biological samples are recorded and presented in Fig. 9a–c. Images of *E. coli* and fluorescence signal from CDs are well merged with each other, thus indicating that the prepared CDs can be used as an effective fluorescent label in bio-imaging. Since the prepared CDs featuring high-color-purity (FWHM~24 nm) long-wavelength one-photon and NIR induced two-photon red FL emission, thus, it is highly desirable in the application of fluorescence bio-labeling.

### Fluorescence in vivo imaging in animal models

In vivo imaging experiments are carried out by injecting 100 μL of protonated CDs into the nude mice at concentration of 200 μg·mL^−1^. The experiment is performed using the PerkinElmer in vivo imaging system under the excitation of 560 nm. The results of in vivo gavage imaging are shown in Fig. 9d, e. In vivo fluorescence images of the mouse before and after gavage injection of the CDs are taken after keeping nesteia for 24 h. As shown in Fig. 9d, before gavage injection, there were only weak FL signals in the section of enterocoelia. After gavage injection (see Fig. 9e), the protonated CDs emits bright FL signals in the section of stomach, which strongly confirms that the deep-tissue penetration of the red FL emission from the protonated CDs. The results of subcutaneous in vivo imaging are shown in Supplementary Fig. 13. It can be observed that, when injected with protonated CDs in different subcutaneous positions and illuminated with the excitation light, the bight FL signals of the CDs are detected to originate from different locations in the mouse tissues. The noise-signal ratio of the CDs calculated by the software of PerkinElmer in vivo imaging system is shown as 3.0−3.5 and is reasonable in comparing with other works. There is certainly an opportunity to improve the signal-to-noise ratio, for example, by imaging through the biological tissue with low absorbance and high tissue penetration depth on one hand, while tuning the auto-fluorescence and CD emission, especially in the long-wavelength range, on the other. Furthermore, as shown in the Supplementary Fig. 14, the protonated CDs are revealed to generate the fluorescence (FL) emission in the NIR-II spectral region at 1015 nm under the 600 and 800 nm continuum light excitation. Meanwhile, the in vivo bio-imaging (see Supplementary Fig. 14) confirms that the merits of long-wavelength emission including the low light absorption, weak auto-fluorescence, and deep tissue penetration, rather than only the PL QYs (see Supplementary Table 3), makes our CDs promising for biological applications^8,13^.

## Conclusion

In summary, we devised a facile approach to synthesize CDs featuring one-photon red FL emission at 620 nm and NIR induced two-photon red FL emission at 630 nm. Further investigations reveal that the protonation of newly emerged 2,3-DAPN fluorophore and its interaction with carbon structure decisively changes the molecular state of CDs. In detail, the protonated 2,3-DAPN narrows the photon transition bandgap and finally triggers the one-photon and two-photon red fluorescence emission on CDs with the narrow luminescence band. As the oxidation products of OPD, the emergence of 2,3-diaminophenazine as a PL determiner suggests that fluorophore products of precursor conversion are viable determinants of the desired luminescence properties of CDs. Furthermore, the results also demonstrate that the protonated CDs are highly promising for fluorescence bio-labeling of *Escherichia coli* bacteria and in vivo bio-imaging. Our results open a new way for predicting and controlling PL properties of CDs, which may guide the development of next-generation high-performance photonic devices for a broad range of applications.

## Methods

### Preparation of CDs

1.305 g of sodium sulfate (AR, Shanghai, China) is dissolved in 20 mL of deionized water. Then, 200 mg of OPD (AR, Shanghai, China) were added into the solution of sodium sulfate and further treated with 20 min ultrasound process. After the ultrasonic treatment, 15 mL of the transparent mixture solution were transferred into a polytetrafluoroethylene autoclaves and heated at 220 °C for 6 h. The products were cooled down at room temperature for 24 h, centrifuged at 6000 rpm min^−1^ for 10 min and filtrated through 0.22 μm ultrafiltration membrane. The obtained supernatants were further dialyzed against 2 L deionized water for 12 h and further filtrated through 0.22 μm ultrafiltration membrane. Eventually, clear brown solution of CDs was obtained and freeze-dried.

### Edge protonation treatment of CDs

2 g of CDs were dissolved in 80 mL H_2_SO_4_ aqueous solution (pH 1) and react for 30 min with magnetic stirring. After the edge protonation using pH 1 H_2_SO_4_ solution, the mixture of CDs and H_2_SO_4_ was dialyzed against 2 L deionized water for 6 h with the water refreshed every 2 h. Then, the samples are collected via freeze-drying. To further effectively remove the residual H_2_SO_4_, the samples collected from freeze-drying are washed by deionized water and centrifuging at 16000 rpm·min^−1^ for three times. Finally, the pure protonated CDs are freeze-dried before use.

### Characterization

Particle morphology and particle components are analyzed using transmission electron microscope (TEM, JEOL, JEM-2100F, Japan) and X-ray photoelectron spectroscopy (XPS, AXIS ULTRA DLD, Kratos, Japan), respectively. Two-photon fluorescence (FL) emission of CDs are excited using 808 nm femtosecond laser (Spectra-Physics Laser, Inc, American). Vibrations of optical spectra are recorded by fluorescence spectrophotometer (F-2700 Hitachi, Japan), ultraviolet-visible (UV−Vis) absorption spectrophotometer (Avaspec-2048-2-USB2, Avantes, Netherland), Fourier transform infrared spectrometer (Nicolet 6700, Thermo Scientific, USA), nuclear magnetic resonance spectroscopy (600 MHz, Avance III, Germany), and deep ultraviolet photoelectron spectroscopy (UPS, AXIS ULTRA DLD, Kratos, Japan). Confocal images are taken by using super-resolution multiphoton confocal microscope (Leica TCS SP8 STED 3X).

### Measurement of one-photon FL QYs

The QYs of the produced CDs were measured by using rhodamine B (dissolved in ethanol, QYs = 85%) as a reference.^53^ During the measurements, the CDs were dissolved in 0.1 M H_2_SO_4_ aqueous solution and the CDs were dissolved in deionized water. QYs of the obtained CDs were calculated according to the formula as follows:

1 $${\varPhi }_{{{{{{\rm{sm}}}}}}}={\varPhi }_{{{{{{\rm{st}}}}}}}(\frac{{{{{{\rm{Grad}}}}}}_{{{{{{\rm{sm}}}}}}}}{{{{{{\rm{Grad}}}}}}_{{{{{{\rm{st}}}}}}}})(\frac{{\eta }_{{{{{{\rm{sm}}}}}}}^{2}}{{\eta }_{{{{{{\rm{st}}}}}}}^{2}})$$

where ‘Φ’ stand for QYs, symbol of ‘Grad’ represents the gradient value of integrated fluorescence intensity vs to corresponding absorbance, and ‘η’ denotes the refractive index. Subscript ‘st’ and subscript ‘sm’ are the abbreviations of standard rhodamine B and the prepared CDs samples, respectively. Ultraviolet-Visible (UV−Vis) absorbance of rhodamine B and the CDs were estimated at 550 nm in a 10 mm cuvette, kept below 0.1. The integrated fluorescence intensity is calculated under the area of FL curve.

### Calculation of the HOMO energy level

This calculation is performed following the procedure described elsewhere^34^. The first step is to achieve the linear fit of the region of UPS spectrum that is near the Fermi energy, and then find the point of intersection between the linear fit of the UPS spectrum and the base line. This intersection point indicates the *E*_Fermi_. The second step is to determine the *E*_cutoff_ of the CDs in the similar way as was done for the *E*_Fermi_ in the electron cut-off regions. The third step is to calculate the HOMO energy level of the CDs following the equation: HOMO (eV) = *E*_Incident photon_ – *E*_cutoff_ + *E*_Fermi_.

### Fluorescence bio-labeling of *Escherichia coli*

*Escherichia coli* (ATCC) were purchased from Shanghai Luwei Microbial Co. Ltd and seeded in a confocal petri dish and cultivated at 37 °C for 24 h. Samples of CDs are dissolved in normal saline solutions. Then, the prepared CDs samples were added into Escherichia coli suspension at a final concentration of 400 μg·mL^−1^. Subsequently, the *E. coli* treated with CDs were further cultivated at 37 °C for 6 h, centrifugated at 7000 rpm for 8 min, and washed with normal saline for three times. Finally, the *E. coli* were resuspended and observed under confocal microscope (Leica TCS SP8 STED 3X).

### Fluorescence in vivo imaging of mice

Nude mice (BKL:BALB/c-nu/nu) of specific pathogen-free were purchased from Shang Lab. Animal Research Center. In vivo imaging, experiments are carried out by injecting 100 μL of protonated CDs into the nude mice at a concentration of 200 μg·mL^−1^. The in vivo imaging experiment are performed using the PerkinElmer in vivo imaging system under excitation of 560 nm. The FL signal interference was removed by stopping the food intake of the mice for 24 h before the vein injection. Then the images of the mice were taken before and after the vein injection of CDs by the NIR-II in vivo image system in the same condition.

## Supplementary information

Supplementary Information New

## Data Availability

The data that support the findings in the Supplementary Information has been deposited in the zenodo database under accession code [10.5281/zenodo.5593848]. Further information can be obtained from the corresponding author upon a reasonable request.
