# Supplementary material for: Photoluminescence mechanism of carbon dots: triggering high-color-purity red fluorescence emission through edge amino protonation
Source: Nat Commun. 2021 Nov 25;12:6856. doi: 10.1038/s41467-021-27071-4 (PMC8616937; doi:10.1038/s41467-021-27071-4)
Supplement: Supplementary file 1 — Supplementary Information New [file 41467_2021_27071_MOESM1_ESM.pdf]

## Supplementary Information (SI)

# Photoluminescence Mechanism of Carbon Dots: Triggering High-colour-purity Red Fluorescence Emission through edge amino protonation

*Qing Zhang[1,2]*, Ruoyu Wang[1], Bowen Feng[1], Xiaoxia Zhong[1]*, Kostya (Ken) Ostrikov[3]*

[1]State Key Laboratory of Advanced Optical Communication Systems and Networks, Key Laboratory for Laser Plasmas (Ministry of Education), School of Physics and Astronomy, Shanghai Jiao Tong University, Shanghai 200240, China.

[2]Institute of Molecular Medicine, Renji Hospital, School of Medicine, Shanghai Jiao Tong University, Shanghai 200127, China.

[3]School of Chemistry and Physics and QUT Centre for Materials Science, Queensland University of Technology (QUT), Brisbane QLD 4000, Australia.

E-mail: qingzhang@sjtu.edu.cn; xxzhong@sjtu.edu.cn.

**Table Caption：**

**Supplementary Table 1**. Comparison of photoluminescence red-shift mechanism of multi-color CDs between previous research and our work.

**Supplementary Table 2**. FL QYs of CDs, and 2,3-DAPN molecular.

**Figure Caption：**

**Supplementary Fig. 1 |** Raman spectra of CDs

**Supplementary Fig. 2 |** Zoomed $^{13}C$-NMR spectra of the CDs.

**Supplementary Fig. 3 |** Zoomed $^{1}H$-NMR spectra of the CDs.

**Supplementary Fig. 4 |** $^{13}C$-NMR spectra of 2,3-diaminophenazine (2,3-DAPN), Modified from "2,3-diaminophenazine is the product from the horseradish peroxidase-catalyzed oxidation of o-phenylenediamine" with permission from Elsevier.[17]

**Fig. 5** | $^{1}H$-NMR spectra of 2,3-DAPN, Modified from "2,3-diaminophenazine is the product from the horseradish peroxidase-catalyzed oxidation of o-phenylenediamine" with permission from Elsevier.[17]

**Supplementary Fig. 6** | (a)-(b) UV-vis absorption of OPD, 2,3-DAPN and CDs with or without protonation treatment.

**Supplementary Fig. 7** |(a), (b) FL spectra of protonated OPD kept at deionized water for 6 hours excited by 480 nm and 808 nm femtosecond laser. (c), (d) $^{1}H$-NMR and zoomed $^{1}H$-NMR spectra of OPD molecular kept at deionized water for 6 hours. Due to the oxidation reaction, part of OPD molecular has converted into 2,3-DAPN analogues.

**Supplementary Fig. 8** | (a), (b) UV-vis absorption of OPD with or without protonation treatment; (c), (d) FL emission of CDs, OPD and 2,3-DAPN samples with or without protonation treatment the identical excitation conditions.

**Supplementary Fig. 9** | (a), (c) Up-conversion FL spectra of 2,3-DAPN at pH7 and pH 1 under 808 nm femtosecond laser excitation. (b), (d) Relationship of FL intensity and femtosecond (fs) laser power at pH 7 and pH 1.

**Supplementary Fig. 10** | (a), (c) Up-conversion FL spectra of OPD at pH7 and pH 1 under 808 nm femtosecond (fs) laser excitation. (b), (d) Relationship of FL intensity and fs laser power at pH 7 and pH 1.

**Supplementary Fig. 11** | (a), (b), and (c) Transmission electron microscopy imaging (TEM), high resolution image and Size distribution of CDs; (d), (g) UV-vis absorption spectra of CDs with or without protonation; (e), (h) FL spectra of CDs dissolved without protonation (excited at 400 nm) and with protonation (excited at 560 nm); (f), (i) FL excitation spectra of CDs with or without protonation.

**Supplementary Fig. 12** | (a)-(d) Raman spectra of CDs prepared at 60,120, 180 and 220℃. (e) and (f) FL emission of CDs synthesized with or without protonation.

**Supplementary Fig. 13** | (a) and (b) FL emission of the protonated CDs excited under 600 and 800 nm continuum light source; (c) and (d) NIR-II in vivo images of mice before and after vein injection.

**Supplementary Fig. 14** | (a) Photograph of LED device fabricated using CDs; (b) FL emission spectrum of the LED device excited by UV light-emitting chips; (c) Color coordinates picture of the LED device (0.26, 0.59).

## *Section 1: Supplementary Information of Tables.*

**Supplementary Table 1**. Comparison of photoluminescence red-shift mechanism of multi-color CDs between previous research and our work.

| Reference | Journal | Mechanism of PL red-shift |
|---|---|---|
| 1[1] | Carbon 2014, 70, 279. | Increasing degree of $COO^-$ |
| 2[2] | Advanced Materials 2015, 27, 1663 | Increasing degree of oxidation |
| 3[3] | Angew. Chem. - Int. Ed. 2015, 54, 2970 | Increasing degree C-O-C & C-O |
| 4[4] | Acs Nano 2016, 10, 484 | Increasing degree of COO- |
| 5[5] | Nanoscale 2016, 8, 729 | Increasing degree of N content |
| 6[6] | ACS Nano 2017, 11, 12402. | Increasing degree of graphitic-N |
| 7[7] | Green Chem. 2017, 19, 3611. | Increasing degree of oxidation |
| 8[8] | Advanced Materials, 2018, 30. | Increasing degree of graphitization and surface modification of -COOH |
| Our work | | Protonation of 2,3-Diaminophenazine |

**Supplementary Table 2**. FL QYs of CDs, and 2,3-DAPN molecular.

| QYs | CDs | 2,3-DAPN |
|---|---|---|
| Without protonation | 14% | 24% |
| With protonation | 1.9% | 0.2% |

**Supplementary Table 3**. FL QYs of CDs, and 2,3-DAPN molecular.

| **Reference** | **NIR Organic dye** | **QYs (%)** |
|---|---|---|
| 1 | IR-1061 (Commercial dye) | **1.7%**[9] |
| 2 | LZ1105 | **1.6%**[10] |
| 2 | IR-E1 | **0.7%**[11] |
| 3 | IR-FTP | **0.02%**[12] |
| 4 | IR-FTTP | **0.1%**[13] |
| 5 | CH1055-PEG | **0.3%**[14] |
| 6 | CH-4T | **1.1%**[15] |
| 7 | SCH1100 | **0.2%**[16] |

## *Section 2: Supplementary Figures*

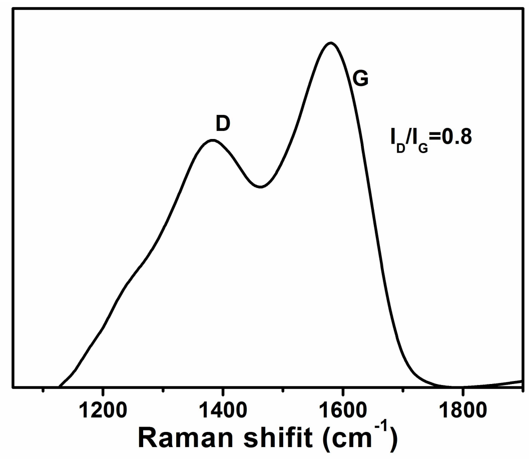


**Supplementary Fig. 1** Raman spectra of CDs.

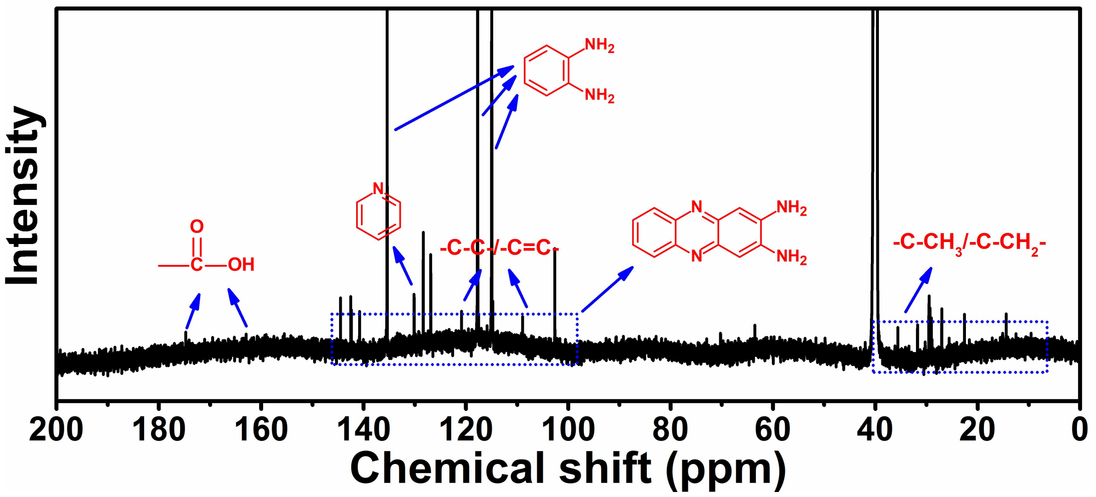


**Supplementary Fig. 2 |** Zoomed $^{13}$C-NMR spectra of the CDs.

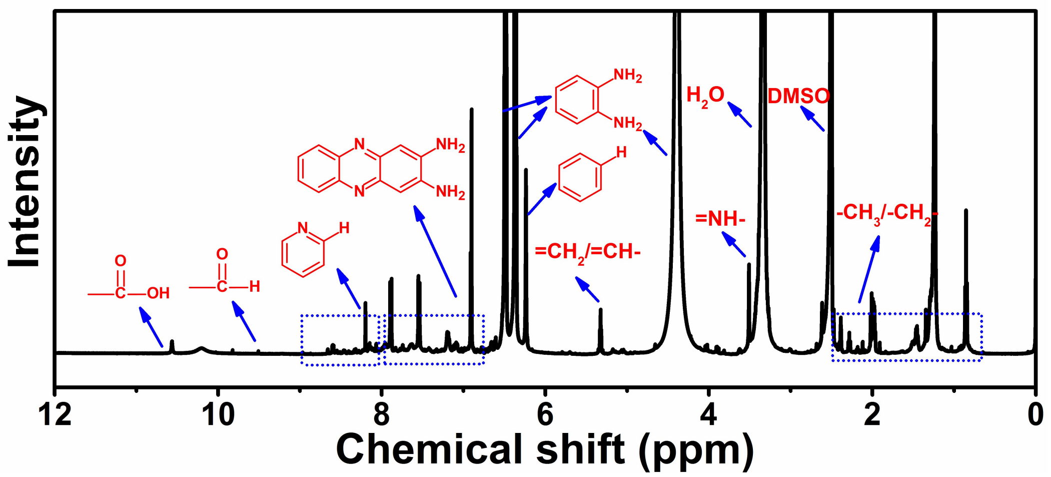


**Supplementary Fig. 3|** Zoomed $^{1}$H-NMR spectra of the CDs.

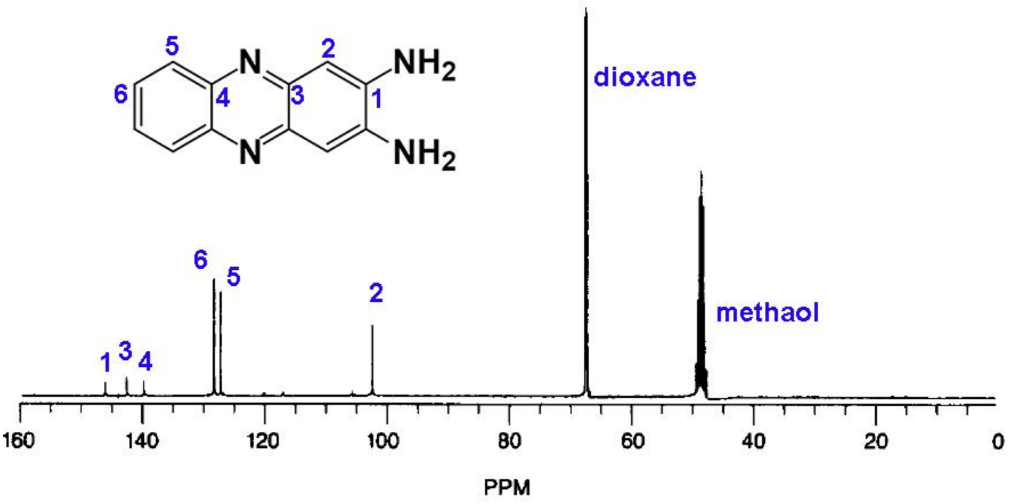


**Supplementary Fig. 4 |** $^{13}$C-NMR spectra of 2,3-diaminophenazine (2,3-DAPN), Modified from "2,3-diaminophenazine is the product from the horseradish peroxidase-catalyzed oxidation of o-phenylenediamine" with permission from Elsevier [17].

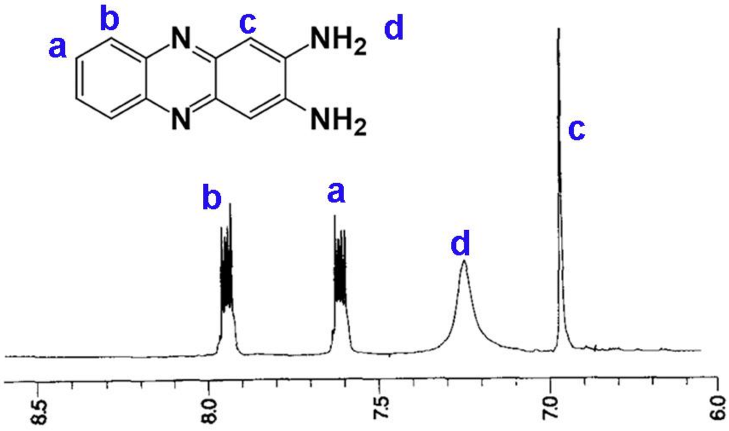


**Supplementary Fig. 5 |** $^1$H-NMR spectra of 2,3-DAPN, Modified from "2,3-diaminophenazine is the product from the horseradish peroxidase-catalyzed oxidation of o-phenylenediamine" with permission from Elsevier[17].

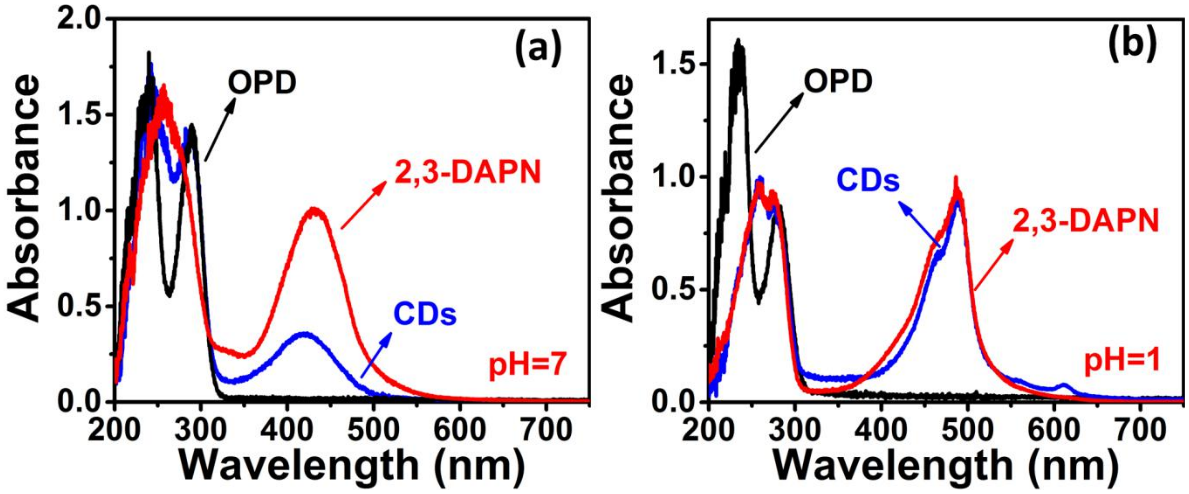


**Supplementary Fig. 6** | (a)-(b) UV-vis absorption of OPD, 2,3-DA PN and CDs with or without protonation treatment.

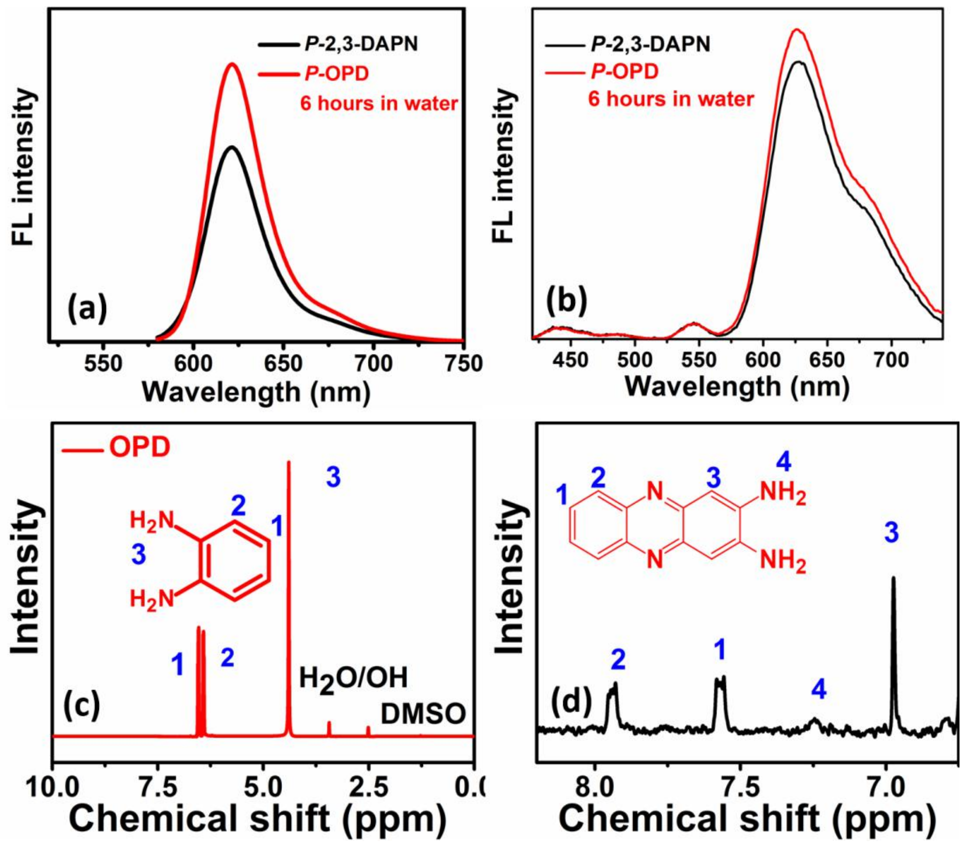


**Supplementary Fig. 7 |** (a), (b) FL spectra of the protonated OPD kept in deionized water for 6 hours excited by 480 nm and 808 nm femtosecond laser. (c), (d) $^{1}$H-NMR and magnified $^{1}$H-NMR spectra of the OPD molecular kept in deionized water for 6 hours. Due to the oxidation reaction, a part of the OPD molecular has converted into 2,3-DAPN analogues.

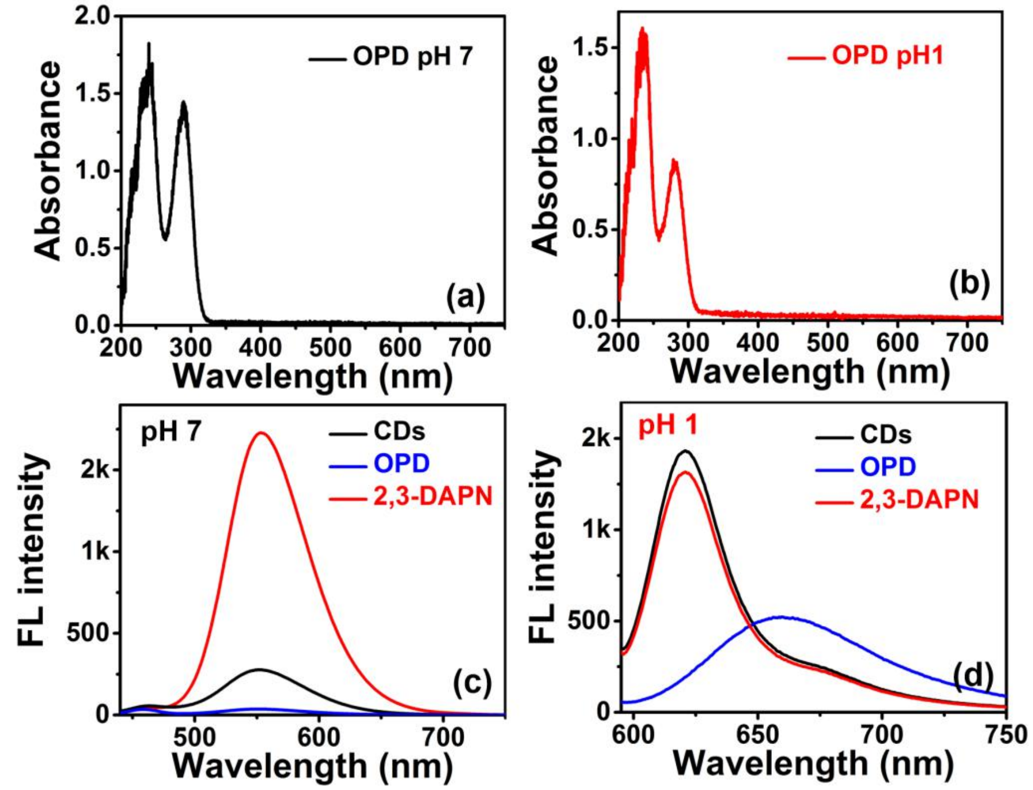


**Supplementary Fig. 8** | (a), (b) UV-vis absorption of OPD with or without protonation treatment; (c), (d) FL emission of CDs, OPD and 2,3-DAPN samples with or without protonation treatment the identical excitation conditions.

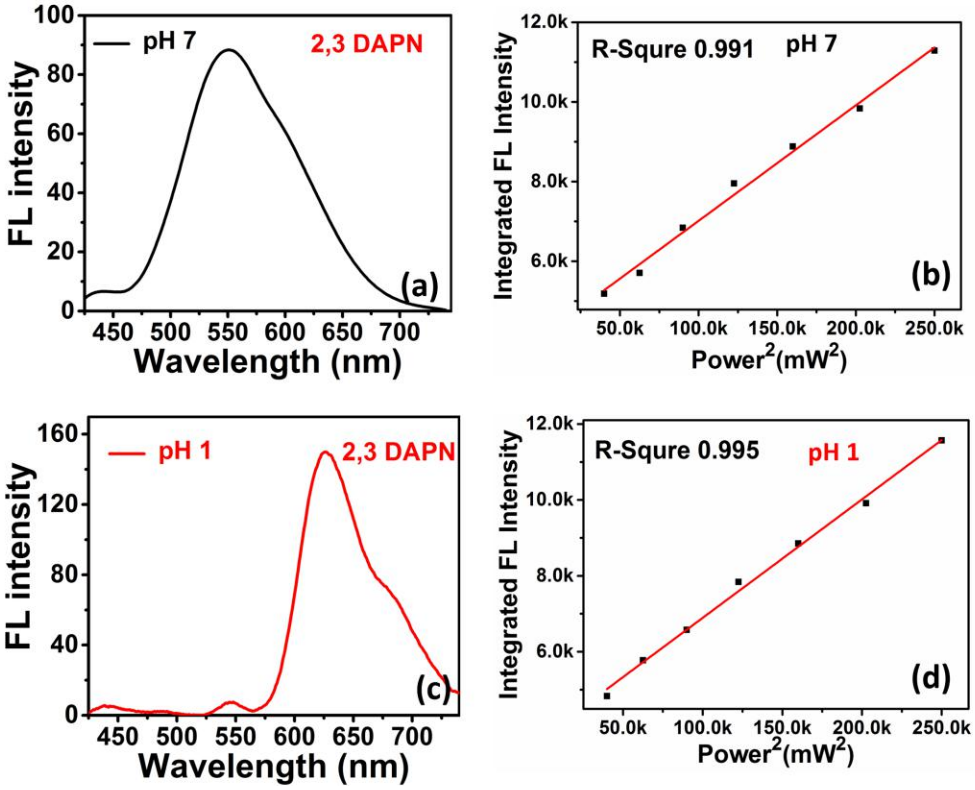


**Supplementary Fig. 9** | (a), (c) Up-conversion FL spectra of 2,3-DAPN at pH7 and pH 1 under 808 nm femtosecond laser excitation. (b), (d) Relationship of FL intensity and femtosecond (fs) laser power at pH 7 and pH 1.

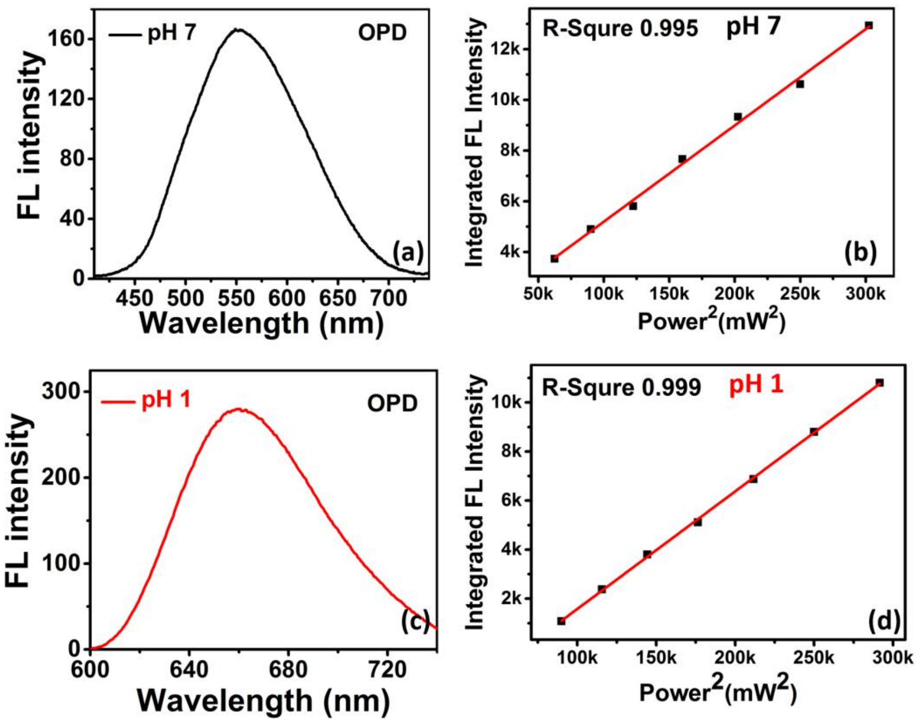


**Supplementary Fig. 10** | (a), (c) Up-conversion FL spectra of OPD at pH7 and pH 1 under 808 nm femtosecond (fs) laser excitation. (b), (d) Relationship of FL intensity and fs laser power at pH 7 and pH 1.

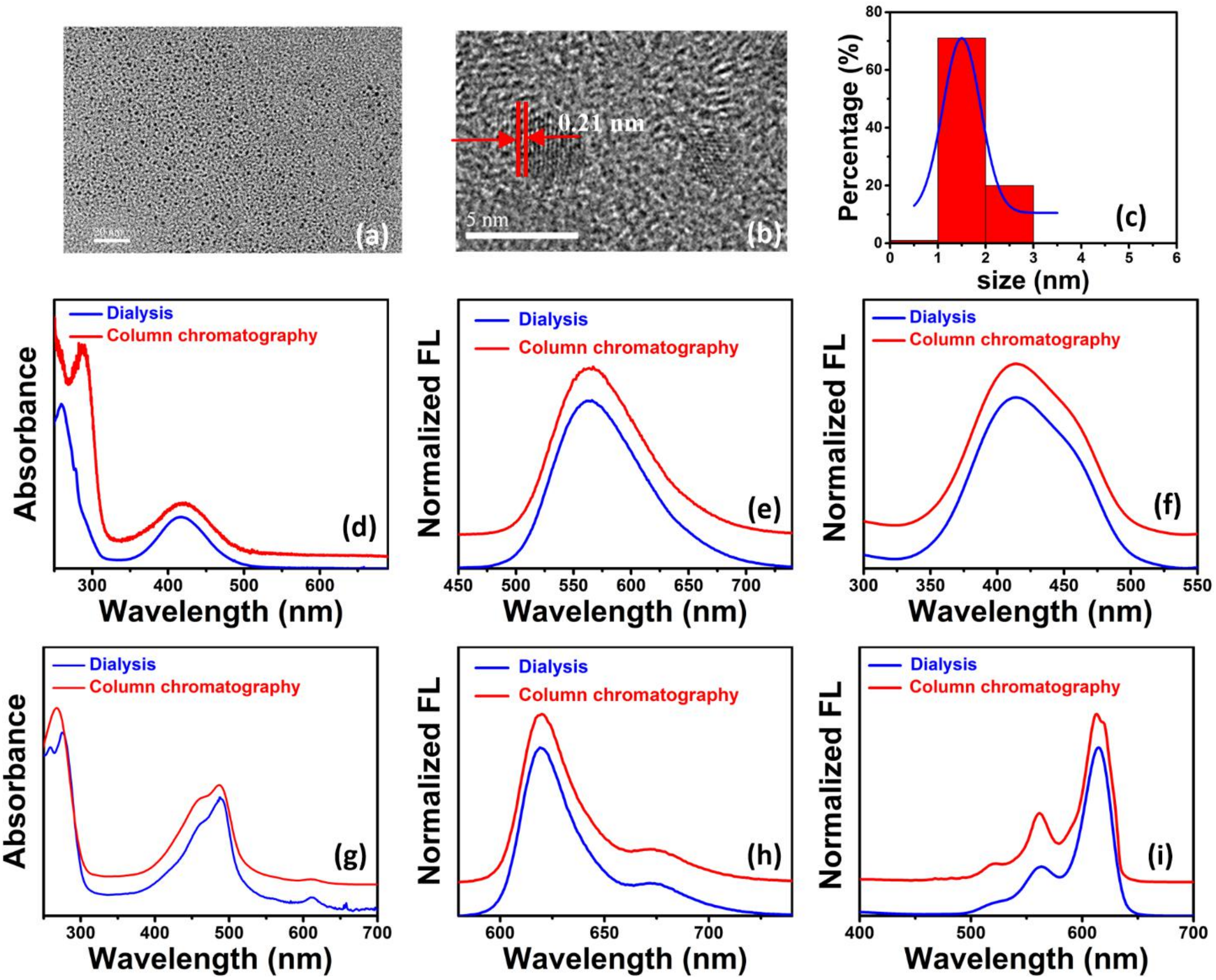


**Supplementary Fig. 11 |** (a), (b), and (c) Transmission electron microscopy imaging (TEM), high resolution image and size distribution of CDs; (d), (g) UV-vis absorption spectra of CDs with or without the protonation; (e), (h) FL spectra of CDs dissolved without the protonation (excited at 400 nm) and with the protonation (excited at 560 nm); (f), (i) FL excitation spectra of CDs with or without the protonation.

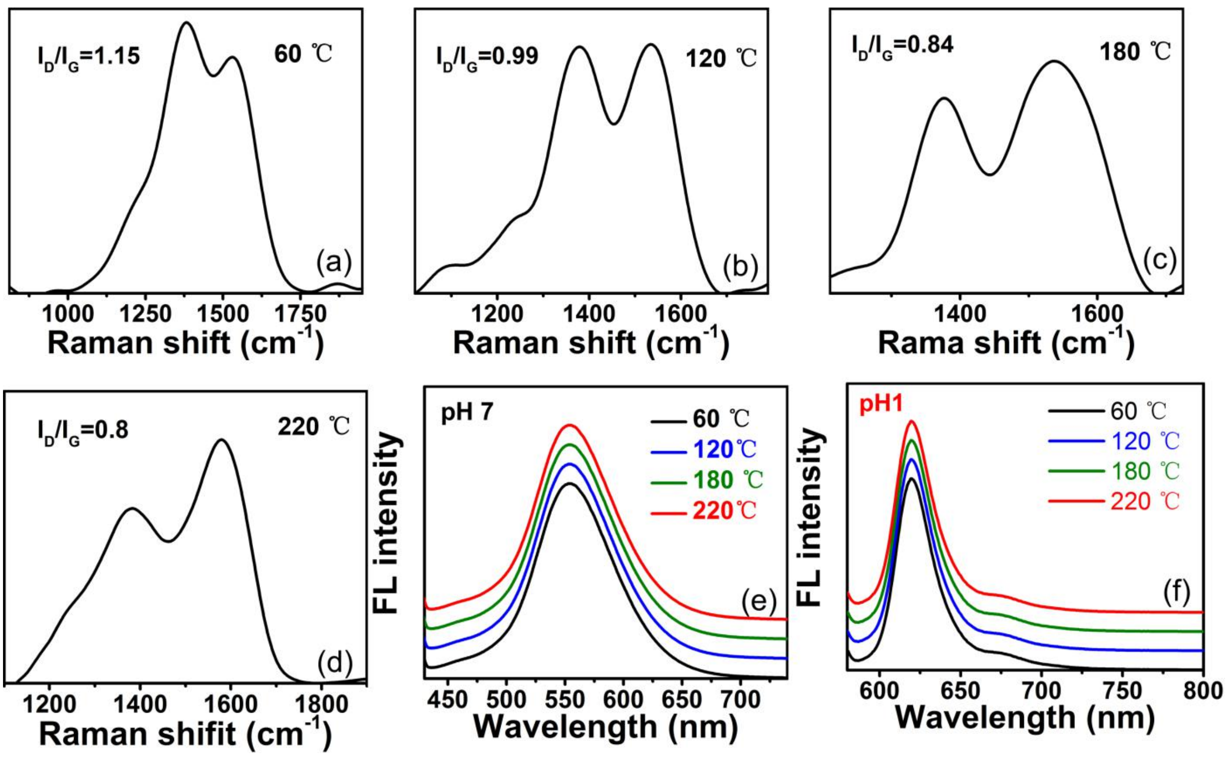


**Supplementary Fig. 12 |** (a)-(d) Raman spectra of CDs prepared at 60,120, 180 and 220℃. (e) and (f) FL emission of CDs synthesized with or without protonation.

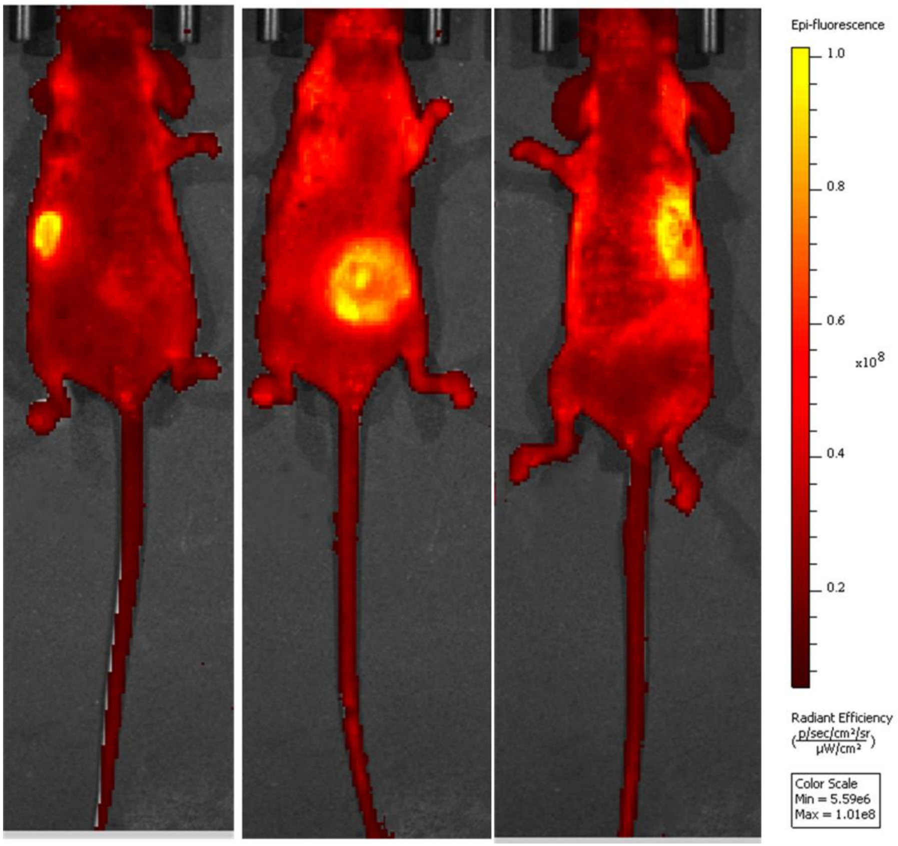


**Supplementary Fig. 13** | In vivo fluorescence images of nude mice injected with different subcutaneous location.

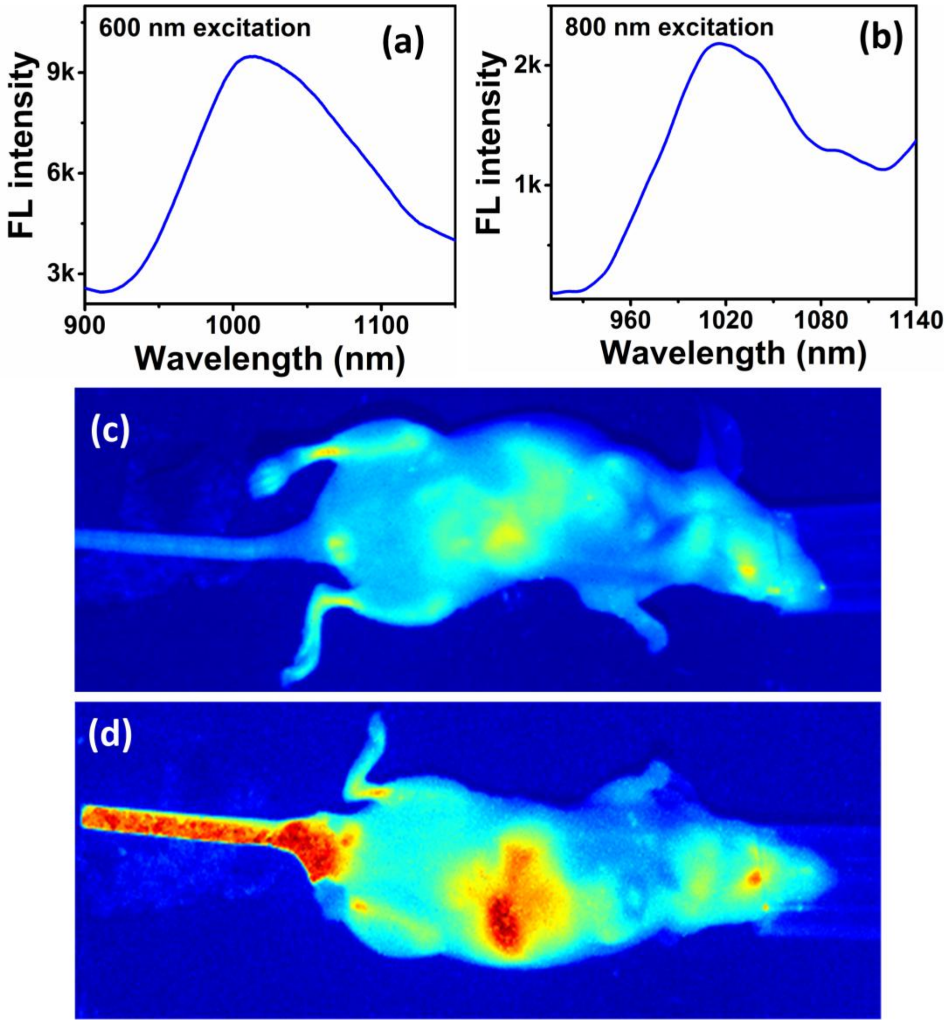


**Supplementary Fig. 14** | (a) and (b) FL emission of the protonated CDs excited under 600 and 800 nm continuum light source; (c) and (d) NIR-II in vivo images of mice before and after vein injection.

## Reference


1. Hola K, *et al.* Photoluminescence effects of graphitic core size and surface functional groups in carbon dots: COO- induced red-shift emission. *Carbon* **70**, 279-286 (2014).
2. Bao L, Liu C, Zhang ZL, Pang DW. Photoluminescence-Tunable Carbon Nanodots: Surface-State Energy-Gap Tuning. *Adv Mater* **27**, 1663-+ (2015).
3. Hu SL, Trinchi A, Atkin P, Cole I. Tunable Photoluminescence Across the Entire Visible Spectrum from Carbon Dots Excited by White Light. *Angew Chem-Int Edit* **54**, 2970-2974 (2015).
4. Ding H, Yu S-B, Wei J-S, Xiong H-M. Full-Color Light-Emitting Carbon Dots with a Surface-State-Controlled Luminescence Mechanism. *Acs Nano* **10**, 484-491 (2016).
5. Guo L*, et al.* Tunable Multicolor Carbon Dots Prepared from Well-defined Polythiophene Derivatives and their Emission Mechanism. **8**, 729 (2015).
6. Hola K*, et al.* Graphitic Nitrogen Triggers Red Fluorescence in Carbon Dots. **11**, 12402-12410 (2017).
7. Liu ML, Yang L, Li R, Chen BB, Liu H, Huang CZ. Large-scale simultaneous synthesis of highly photoluminescent green amorphous carbon nanodots and yellow crystalline graphene quantum dots at room temperature. **19**, 10.1039.C1037GC01236E (2017).
8. Miao X*, et al.* Synthesis of Carbon Dots with Multiple Color Emission by Controlled Graphitization and Surface Functionalization. *Adv Mater* **30**, (2018).
9. Tao Z*, et al.* Biological Imaging Using Nanoparticles of Small Organic Molecules with Fluorescence Emission at Wavelengths Longer than 1000 nm. *Angew Chem*, e201307346 (2013).
10. Li BH*, et al.* Organic NIR-II molecule with long blood half-life for in vivo dynamic vascular imaging. *Nat Commun* **11**, (2020).
11. Zhang XD*, et al.* Traumatic Brain Injury Imaging in the Second Near-Infrared Window with a Molecular Fluorophore. *Adv Mater*, 6872-6879 (2016).
12. Yang*, et al.* Rational Design of Molecular Fluorophores for Biological Imaging in the NIR-II Window. *Adv Mater*, e1605497.
13. Yang Q, Hu Z, Zhu S, Rui M, Dai H. Donor Engineering for NIR-II Molecular Fluorophores with Enhanced Fluorescent Performance. *J Am Chem Soc* **140**, 1715-1724 (2018).
14. Antaris AL*, et al.* A small-molecule dye for NIR-II imaging. *Nat Mater*, 235-242 (2016).
15. Antaris AL*, et al.* A high quantum yield molecule-protein complex fluorophore for near-infrared II imaging. *Nat Commun* **8**, 15269 (2017).
16. Novel benzo-bis(1,2,5-thiadiazole) fluorophores for in vivo NIR-II imaging of cancer. *Chem Sci*, 6203-6207 (2016).
17. Tarcha PJ, Chu VP, Whittern D. 2,3-diaminophenazine is the product from the horseradish peroxidase-catalyzed oxidation of o-phenylenediamine. *Anal Biochem* **165**, 230-233.
